# Autism-Associated DNA Methylation at Birth From Multiple Tissues Is Enriched for Autism Genes in the Early Autism Risk Longitudinal Investigation

**DOI:** 10.3389/fnmol.2021.775390

**Published:** 2021-11-25

**Authors:** Kelly M. Bakulski, John F. Dou, Jason I. Feinberg, Max T. Aung, Christine Ladd-Acosta, Heather E. Volk, Craig J. Newschaffer, Lisa A. Croen, Irva Hertz-Picciotto, Susan E. Levy, Rebecca Landa, Andrew P. Feinberg, Margaret D. Fallin

**Affiliations:** ^1^Department of Epidemiology, School of Public Health, University of Michigan, Ann Arbor, MI, United States; ^2^Department of Mental Health, Bloomberg School of Public Health, Johns Hopkins University, Baltimore, MD, United States; ^3^Wendy Klag Center for Autism and Developmental Disabilities, Baltimore, MD, United States; ^4^Center for Epigenetics, Johns Hopkins School of Medicine, Baltimore, MD, United States; ^5^Department of Biostatistics, School of Public Health, University of Michigan, Ann Arbor, MI, United States; ^6^Department of Epidemiology, Johns Hopkins Bloomberg School of Public Health, Johns Hopkins University, Baltimore, MD, United States; ^7^College of Health and Human Development, Penn State University, State College, PA, United States; ^8^Kaiser Permanente Division of Research, Oakland, CA, United States; ^9^Department of Public Health Sciences, School of Medicine, University of California, Davis, Davis, CA, United States; ^10^MIND Institute, University of California, Davis, Davis, CA, United States; ^11^Children’s Hospital of Philadelphia, Philadelphia, PA, United States; ^12^Kennedy Krieger Institute Center for Autism and Related Disorders, Baltimore, MD, United States; ^13^Department of Medicine, School of Medicine, Johns Hopkins University, Baltimore, MD, United States; ^14^Department of Biostatistics, Bloomberg School of Public Health, Johns Hopkins University, Baltimore, MD, United States

**Keywords:** autism spectrum disorder, DNA methylation, biomarker, epidemiology, cord blood

## Abstract

**Background:** Pregnancy measures of DNA methylation, an epigenetic mark, may be associated with autism spectrum disorder (ASD) development in children. Few ASD studies have considered prospective designs with DNA methylation measured in multiple tissues and tested overlap with ASD genetic risk loci.

**Objectives:** To estimate associations between DNA methylation in maternal blood, cord blood, and placenta and later diagnosis of ASD, and to evaluate enrichment of ASD-associated DNA methylation for known ASD-associated genes.

**Methods:** In the Early Autism Risk Longitudinal Investigation (EARLI), an ASD-enriched risk birth cohort, genome-scale maternal blood (early *n* = 140 and late *n* = 75 pregnancy), infant cord blood (*n* = 133), and placenta (maternal *n* = 106 and fetal *n* = 107 compartments) DNA methylation was assessed on the Illumina 450k HumanMethylation array and compared to ASD diagnosis at 36 months of age. Differences in site-specific and global methylation were tested with ASD, as well as enrichment of single site associations for ASD risk genes (*n* = 881) from the Simons Foundation Autism Research Initiative (SFARI) database.

**Results:** No individual DNA methylation site was associated with ASD at genome-wide significance, however, individual DNA methylation sites nominally associated with ASD (*P* < 0.05) in each tissue were highly enriched for SFARI genes (cord blood *P* = 7.9 × 10^–29^, maternal blood early pregnancy *P* = 6.1 × 10^–27^, maternal blood late pregnancy *P* = 2.8 × 10^–16^, maternal placenta *P* = 5.6 × 10^–15^, fetal placenta *P* = 1.3 × 10^–20^). DNA methylation sites nominally associated with ASD across all five tissues overlapped at 144 (29.5%) SFARI genes.

**Conclusion:** DNA methylation sites nominally associated with later ASD diagnosis in multiple tissues were enriched for ASD risk genes. Our multi-tissue study demonstrates the utility of examining DNA methylation prior to ASD diagnosis.

## Background

Autism spectrum disorder (ASD) is a complex neurodevelopmental disorder estimated to affect one in 54 US children by age 8 years (Maenner et al., 2020). ASD is characterized by deficits in communication, social interaction, and behavioral flexibility (King et al., 2014). ASD neuropathology likely begins during the *in utero* period, as evidenced by neuroanatomy (Rodier et al., 1996; Bailey et al., 1998; Bauman and Kemper, 2005), teratogen exposure timing (Stromland et al., 1994; Williams et al., 2001), and behavior differences observed in very early life (Zwaigenbaum et al., 2005). Children with ASD are estimated to require an additional $17,081 more support per year, based on the combined needs related to health care, school services, ASD-related therapies, family coordinated services, and caregiver time (Lavelle et al., 2014). ASD symptomology at 36-months may be improved with intervention during the first year of life (Rogers et al., 2014), and some preschoolers with intervention also display reduced ASD outcomes (National Research Council, 2001). Participation in these intervention programs depends on early diagnosis, which is often difficult (Chawarska et al., 2014). Identifying early biomarkers for those at enriched risk of ASD is an important area of research.

Autism spectrum disorder heritability estimates indicate the etiology is a combination of heritable and environmental factors (Tick et al., 2016; Sandin et al., 2017). Inherited and *de novo* genetic risk factors for ASD have been identified from several large genome-wide scans (Consortium, 2017), though a proportion of the genetic risk has not yet been explained (Miles, 2011). Likewise, potential prenatal toxicant risk factors have been identified, but more research is needed (Rossignol et al., 2014). Genetic and environmental factors likely interact to contribute to risk (Cheroni et al., 2020). Epigenetics broadly represent information for gene regulation, not carried in the genetic sequence itself (Greally, 2018). Epigenetic marks, such as DNA methylation of cytosine nucleotides, have been associated with both genetic variation and environmental vulnerability, making them a potential mechanisms of either type of risk, or their interaction (Bakulski and Fallin, 2014). Altered DNA methylation has been implicated in ASD (Vogel Ciernia and LaSalle, 2016; Hannon et al., 2018; Mordaunt et al., 2020), including ASD neuropathology (Ladd-Acosta et al., 2014). DNA methylation varies by tissue, cell type, and time (Bakulski et al., 2016b), and incorporating data across multiple domains will provide biomarker insights.

Together, placenta and cord blood DNA methylation at birth as well as pregnancy blood DNA methylation have not yet been examined for prospective association with ASD development. In the current study, we test genome-wide DNA methylation patterns in pregnancy (blood) and birth samples (cord blood and placenta tissue) from a high ASD risk birth cohort for association with 36-month ASD phenotypes. We examine DNA methylation globally, at single CpG sites, and across regions. Enrichment of biological processes and ASD risk genes were also tested.

## Materials and Methods

### Study Sample and Phenotype Assessment

The Early Autism Risk Longitudinal Investigation (EARLI) is an enriched autism pregnancy cohort (Newschaffer et al., 2012). This longitudinal study recruited mothers of children with confirmed ASD who were early in a subsequent pregnancy or were trying to become pregnant. Maternal participants had to live within 2 h of a study site (Philadelphia, Baltimore, San Francisco/Oakland, and Sacramento), be at least 18 years of age, be competent to communicate in English, and be no more than 28 weeks pregnant. There were 232 mothers participating in the study. The EARLI study was reviewed and approved by Human Subjects Institutional Review Boards (IRBs) from each of the four study sites (Drexel University, Johns Hopkins University, University of California Davis, and Kaiser Permanente Research), as well as the secondary analysis site at the University of Michigan.

At the 36-month study visit, EARLI siblings participated in a clinician administered neurological assessment that included consensus ASD diagnosis using the Diagnostic and Statistical Manual of Mental Disorders (DSM-5). During this study visit, measures were taken with the Social Responsiveness Scale (SRS), Mullens Scales of Early Learning, and Vineland. EARLI siblings were categorized into three outcome levels (ASD, non-typically developing, and typically developing) according to the previously published Baby Siblings Research Consortium algorithm. Those that met clinical criteria and above the Autism Diagnostic Observation Schedule cutoff were categorized as ASD. Those that did not meet criteria for ASD, but multiple Mullen subsets ≥ 1.5 standard deviations below the mean and/or one Mullen subset ≥ 2 standard deviations below the mean and/or Autism Diagnostic Observation Schedule ≤ 3 points below the ASD cutoff were categorized as non-typically developing. Finally, those that did not meet either of the previous criteria were categorized as typically developing (Ozonoff et al., 2014). The three-level categorization of ASD, non-typically developing, and typically developing at 36 months was the primary phenotype of interest in this study.

### Biological Processing and DNA Methylation Measurement

Cord blood biospecimens were collected with butterfly needles into EDTA tubes and archived at 175 births. Membranes were removed and full thickness placental tissue from a central cotyledon was collected. Sterile punch biopsy forceps were used to extract placental samples from the maternal and fetal sides. Maternal venous blood was collected during pregnancy at two time points (early and late pregnancy) into EDTA tubes. Samples were transported to the Johns Hopkins Biological Repository (JHBR) for aliquotting and archiving (–80°C).

Genomic DNA was extracted from blood using the DNA Blood Midi kit (Qiagen, Valencia, CA, United States) on the QIAsymphony automated workstation with the Blood 1000 protocol, according to manufacturer’s instructions. Placental DNA was extracted with the DNeasy Tissue Kit (Qiagen Sciences, Germantown, MD, United States). DNA was quantified using the Nanodrop (ThermoFisher, Waltham, MA, United States) and normalized DNA aliquots were sent to the Center for Inherited Disease Research (Johns Hopkins University). There, 1-μg DNA samples were bisulfite treated and cleaned using the EZ DNA methylation gold kit (Zymo Research, Irvine, CA, United States) according to manufacturer’s instructions. DNA was plated randomly and was assayed on the Infinium HumanMethylation450 BeadChip (Illumina, San Diego, CA, United States) (Bibikova et al., 2011). Methylation control gradients and between-plate repeated tissue controls were used.

### Bioinformatic and Statistical Approach

In R statistical software (version 3.3), we used the minfi (version 1.22.1) (Aryee et al., 2014). Bioconductor package to process raw Illumina image files into background corrected and noob background corrected values (Triche et al., 2013). Probes with failed detection *P*-value (>0.01) in >10% of samples (*n* = 635) or noted cross-reactive probes (*n* = 29,154) (Chen et al., 2013) were removed. We checked for samples with low overall array intensity (<10.5 relative fluorescence units) and high (>20%) proportion of failed detection *P*-value (*n* = 0). Samples with discordant methylation predicted sex and observed sex were removed (*n* = 3). Principal components of the full methylation data were used to assess potential batch effects, particularly associated with measured variables such as hybridization date, position on the plate, and study site. In each tissue set, we used ANOVA tests to assess potential associations between DNA methylation principal components and the measured covariates, and we visualized the findings with a heatmap of the *P*-values.

For cord blood, cell type proportions for granulocytes, monocytes, B-cells, natural killer cells, CD8+ T-cells, CD4+ T-cells, and nucleated red blood cells were estimated from methylation measurements using signatures from sorted cord blood cell populations (Bakulski et al., 2016a). For maternal blood, an adult blood reference set (Reinius et al., 2012) was used to estimate cell type proportions (Houseman et al., 2012). For placenta, we used a reference of placentas from term pregnancies (Yuan et al., 2021) to estimate placenta cell type proportions. Surrogate variables can efficiently incorporate variability due to known and unknown covariates (Leek and Storey, 2007). Separately in each tissue, we estimated surrogate variables associated with DNA methylation, protecting the relationship between DNA methylation and ASD status using the sva package (Leek and Storey, 2007). Our primary DNA methylation analyses were on the beta-value scale (ranging in values of 0 to 1) and as a sensitivity analysis we examined the *M*-value scale (log_2_ ratio of the methylated and unmethylated intensity).

For all statistical analyses, our primary comparison was cord blood DNA methylation in relation to childhood ASD status, relative to typical development as the reference group. ASD phenotyping data at 36-months were available on 133 children with cord blood DNA methylation measures. Children were classified as typically developing, non-typically developing, or as having ASD. Bivariate relationships between 36-month ASD outcomes and covariates of interest were calculated and a linear trend test of ordinal variables was used to estimate statistical significance.

To assess the tissue specificity of our findings, for secondary analyses we performed parallel analyses of DNA methylation from maternal blood early in pregnancy (*n* = 140), maternal blood late in pregnancy (*n* = 75), placental tissue from the maternal side (*n* = 106) and placental tissue from the fetal side (*n* = 107) with matched 36-month child phenotypes. Sample sizes vary because at each of the study visits and for each of the sample types, slightly different numbers of participants were able to provide samples and have sufficient DNA extracted.

First, we tested for differences in estimated global DNA methylation by ASD status across all autosomal sites and by Illumina annotated genomic region (CpG island, shore, shelf, open sea, and enhancer). For each sample, we estimated global DNA methylation by averaging DNA methylation across all autosomal sites, and we stratified by each region and again took the average (Bakulski et al., 2020). To assess differences in the distributions of global DNA methylation by ASD status overall and across each genomic region, we used linear regression, adjusted for surrogate variables. As a sensitivity analysis, we stratified by child sex and repeated the regressions. We also tested for differences in the DNA methylation cumulative density function across samples, between diagnosis groups (Zhao et al., 2015).

Second, we performed multivariable linear regression at individual probes to identify DNA methylation differences associated with the ASD diagnosis, relative to typical development, adjusted for surrogate variables. The number of surrogate variables chosen to use in models was done by sequentially adding surrogate variables and computing lambda inflation. The model closest to a lambda inflation factor of 1.0 was chosen, with preference for less surrogate variables if multiple models fit our criteria. We used three surrogate variables in all tissues, except for maternal side placenta in which two surrogate variables were chosen. As sensitivity analysis, we also performed tests with adjusting for all estimated surrogate variables in each tissue and compared the effect estimates observed to those from the primary model with Pearson correlation tests. To estimate standard error, empirical Bayesian methods were used (Smyth, 2004). We estimated false discovery rates to account for multiple testing. We visualized results using volcano plots of the magnitude of DNA methylation differences between ASD diagnosis and typically developing with the –log_10_(*P*-value) of that association. Among sites reaching nominal *p*-value < 0.05, we calculated the percent with higher or lower DNA methylation in ASD cases. We tested for correlation of the effect estimates from each of the five tissues using Pearson correlation. As a sensitivity analysis, we tested associations between DNA methylation at individual probes with ASD status, adjusted for known covariates: ancestry principal components, batch, maternal age, maternal education, gestational age (not used in maternal blood models), fetal sex (not used in maternal blood models), granulocyte predicted cell type proportions in blood models (additionally nucleated red blood cell proportion in cord blood), and syncytiotrophoblast and Hofbauer predicted cell type proportions in placenta models. We considered study site as a covariate, but in each of the five tissue sets, it was not associated *(P*-value < 0.05) with principal components of DNA methylation data (Supplementary Figure 1). We compared models using surrogate variables to models with known covariates by calculating Pearson correlation coefficients between the effect estimates. As a sensitivity analysis in cord blood we tested the association between DNA methylation on the *M*-value scale with ASD status, adjusting for covariates as described. We compared these effect estimates and *P*-values with those from models with DNA methylation on the beta-value scale with Pearson correlation coefficients.

Third, we tested the DNA methylation sites associated with ASD for enrichment in SFARI risk genes^1^ (*n* = 881) (Banerjee-Basu and Packer, 2010) using a chi squared test statistic. CpG probes were annotated to genes using the Illumina annotation file. We identified overlapping SFARI genes across the five tissues and visualized the data with a Venn diagram. For testing enrichment of SFARI risk genes, we selected DNA methylation sites associated with ASD using multiple *P*-value criteria (0.0001, 0.001, 0.005, 0.01, 0.02, 0.03, 0.04, 0.05, 0.1, 0.15, 0.2, 0.3, 0.5, 0.7, 0.99). As a negative control, we ran the same analysis in 10 permutations, randomly selecting sets of genes (same number as SFARI set) present on the Illumina annotation. We also restricted enrichment testing in cord blood to CpGs that were meQTL targets to see if enrichment was stronger in these meQTL targets (Andrews et al., 2017).

Fourth, we tested whether the DNA methylation sites associated with ASD were enriched in gene ontology biological processes using Wallenius’ non-central hypergeometric distribution through the missMethyl package (Phipson et al., 2016). Entrez gene identifiers were annotated to CpGs and tested against the array background, while adjusting for the array design. We restricted to a minimum of five genes per term and we used REVIGO to remove redundant gene ontologies (Supek et al., 2011). To assess similarity of gene ontologies across tissues, we took the sum of the ranks of gene ontologies across cord blood, maternal blood at two time points, and two sides of placenta.

Fifth, to assess the specificity of findings to ASD rather than impaired neurodevelopment more broadly, as sensitivity analyses throughout, we also compared the non-typically developing group to the typically developing group. We repeated the previously described analytic pipeline on this sample.

Code to conduct all analyses is available^2^. Data are available through the National Institute of Mental Health Data Archive (NDA) under the collections for the EARLI network (1600, 2462, and 2563).

## Results

### DNA Methylation Data Quality Assessment

Autism spectrum disorder, non-typically developing, and typically developing status did not vary by technical covariates such as round of measurement (*P*-value = 0.9). Replicates across 450k plates (*n* = 14) were highly correlated. Unnormalized DNA methylation Pearson correlation between replicates across plates ranged from 0.848 to 0.996 with a mean (SD) of 0.963 (0.041). After probe and sample filtering, 455,723 CpG sites remained in analysis for 133 cord blood samples, 143 early pregnancy maternal blood samples, 78 late pregnancy maternal blood samples, 101 fetal side placenta samples, and 101 maternal side placenta samples.

### Study Sample Characteristics

Of the 133 EARLI sibling participants with both cord blood DNA methylation data and 36-month neurophenotyping, 29 (21.8%) were diagnosed with ASD, 57 (42.9%) were non-typically developing and 47 (35.3%) were typically developing. Among the ASD cases, 22 (75.9%) were male and this sex ratio significantly differed from the typically developing group, where 44.7% were male (Table 1). Mothers in our sample ranged in age from 21 to 44 and fathers from 22 to 53 years old. Neither maternal nor paternal ages were associated with ASD in our sample. We also did not observe differences in ASD by race/ethnicity, maternal education, or household income. Of the 143 early pregnancy maternal blood samples with neurophenotyping data, 28 (19.6%) had children that were categorized as ASD, 60 (42.0%) as non-typically developing, and 55 (38.5%) as typically developing (Supplementary Table 1). Of the 78 late pregnancy maternal blood samples, 15 (19.2%) had children that were categorized as ASD, 36 (46.2%) as non-typically developing, and 27 (34.6%) as typically developing (Supplementary Table 2). Out of 101 fetal side placenta samples, 18 (17.8%) were from pregnancies of children later categorized as ASD, 48 (47.5%) categorized as non-typically developing, and 35 (34.7%) as typically developing (Supplementary Table 3). Out of 101 maternal side placenta samples, 15 (14.9%) were from pregnancies of children later categorized as ASD, 48 (47.5%) categorized as non-typically developing, and 38 (37.6%) as typically developing (Supplementary Table 4).

**TABLE 1:** Cord blood study sample descriptive statistics by Baby Siblings Research Consortium algorithm typically developing, non-typically developing, and autism spectrum disorder (ASD) categorization.

**Covariate**	**Overall**	**Typically developing *N* = 47**	**Non-typically developing *N* = 57**	**ASD *N* = 29**	***P***
**Sibling sex**					
Female	63 (47.4%)	26 (55.3%)	30 (52.6%)	7 (24.4%)	0.017
Male	70 (52.6%)	21 (44.7%)	27 (47.4%)	22 (75.9%)	
**Maternal age**					
Continuous	33.6 (4.73)	34.4 (4.87)	32.9 (4.81)	33.8 (4.27)	0.29
**Paternal age**					
Continuous	35.3 (5.83)	35.7 (5.93)	34 (5.77)	35.4 (5.93)	0.78
Missing	2 (1.5%)			2 (6.9%)	
**Race/Ethnicity**					
Non-hispanic white	66 (49.6%)	30 (63.8%)	23 (40.4%)	13 (44.8%)	0.65
Non-hispanic black	13 (9.8%)	1 (2.1%)	8 (14.0%)	4 (13.8%)	
Hispanic/latino	20 (15.0%)	7 (14.9%)	7 (12.3%)	6 (20.7%)	
Other	30 (22.6%)	7 (14.9%)	17 (29.8%)	6 (20.7%)	
Missing	4 (3.0%)	2 (4.3%)	2 (3.5%)	0 (0.0%)	
**Maternal education**					
High school or less	13 (9.7%)	3 (6.38%)	5 (8.77%)	5 (17.2%)	0.15
Some college or Associates degree	46 (34.6%)	12 (25.5%)	24 (42.1%)	10 (34.5%)	
Bachelors degree	38 (28.6%)	15 (31.9%)	18 (31.6%)	5 (17.2%)	
Graduate degree	34 (25.6%)	17 (36.2%)	10 (17.5%)	7 (24.1%)	
Missing	2 (1.5%)			2 (6.9%)	
**Household income**					
<$50,000	30 (22.6%)	8 (17.0%)	18 (31.6%)	4 (13.8%)	0.16
50,000–$99,999	45 (33.8%)	17 (36.2%)	15 (26.3%)	13 (44.8%)	
>$100,000	53 (39.8%)	21 (44.7%)	23 (40.4%)	9 (31.0%)	
Missing	5 (3.8%)	1 (2.1%)	1 (1.8%)	3 (10.3%)	
**Batch**					
1	87 (65.4%)	31 (66.0%)	37 (64.9%)	19 (65.5%)	0.99
2	46 (34.6%)	16 (34.0%)	20 (35.1%)	10 (34.5%)	
**Study site**					
Drexel	34 (25.6%)	20 (42.6%)	8 (14.0%)	6 (20.7%)	0.003
Johns Hopkins	33 (24.8%)	4 (8.5%)	20 (35.1%)	9 (31.0%)	
Kaiser	40 (30.1%)	11 (23.4%)	21 (36.8%)	8 (27.6%)	
UC Davis	26 (19.5%)	12 (25.5%)	8 (14.0%)	6 (20.7%)	
**Cell type percent**					
Granulocyte	43.0 (13.0)	43.5 (10.8)	44.4 (14.5)	39.7 (13.0)	0.29
CD8+ Tcell	13.4 (4.3)	13.8 (4.6)	12.6 (4.0)	14.2 (4.1)	0.18
CD4+ Tcell	19.1 (8.2)	18.4 (6.1)	18.9 (8.7)	20.7 (9.9)	0.48
NK cell	0.48 (1.1)	0.59 (1.18)	0.44 (1.17)	0.39 (1.02)	0.69
Bcell	11.0 (3.9)	10.8 (3.8)	11.3 (4.1)	10.8 (3.9)	0.79
Monocyte	8.3 (2.4)	8.9 (2.3)	8.0 (2.6)	8.1 (3.4)	0.12
Nucleated red blood cell	10.1 (5.0)	9.4 (4.3)	9.9 (4.3)	11.5 (7.0)	0.20

*Mean (SD) for continuous covariates and N(%) for categorical covariates.*

### Global DNA Methylation and 36-Month Autism Spectrum Disorder Diagnosis

We examined the global associations across all 455,723 CpG sites, as well as broken up by genomic region: CpG islands (CpG sites *n* = 143,477), shores (*n* = 106,473), shelves (*n* = 43,469), and open sea (*n* = 162,334) (Supplementary Figure 2). Adjusted for surrogate variables, percent DNA methylation averaged across all sites was 0.056 higher in cord blood with ASD (*P*-value = 0.57), 0.062 higher for ASD cases in early pregnancy maternal blood (*P*-value = 0.21), 0.18 higher for ASD cases in late pregnancy maternal blood (*P*-value = 0.03), and 0.034 higher for ASD cases in maternal placenta tissue (*P*-value = 0.78). Fetal placenta tissue had 0.27 lower percent DNA methylation with ASD *P* = 0.02), most pronounced in open sea (–0.39%, *P*-value = 0.006) and shelf (–0.43%, *P* = 0.005) regions. As a sensitivity analysis, we analyzed global DNA methylation differences in cord blood by ASD status, stratified by sex (Supplementary Figure 3). As in the non-stratified analyses, we observed no difference by ASD status, though the direction of effect was in opposite directions for each sex. Results using the cumulative density function global test mirrored these results (Supplementary Table 5). The cumulative densities of DNA methylation were most associated with ASD status in placental tissue (*P*-value = 0.014), particularly in open sea (*P*-value = 0.005) and shelf (*P*-value = 0.008) regions.

### Single Site DNA Methylation and 36-Month Autism Spectrum Disorder Diagnosis

Cord blood DNA methylation models adjusted for three surrogate variables had a lambda inflation factor of 0.99 (Supplementary Figure 4). Surrogate variables were associated with measured covariates such as cell type proportions, baby’s sex, family income, and maternal age (Supplementary Figure 5). In early pregnancy maternal blood, the three surrogate variable model had a lambda of 0.99, and in late pregnancy maternal blood three surrogate variables also reached lambda of 0.99. In the two placenta models, the fetal side model used three surrogate variables for a lambda of 1.02, and the maternal side model used two surrogate variables for a lambda of 1.04 (Supplementary Figure 6).

**FIGURE 1:**
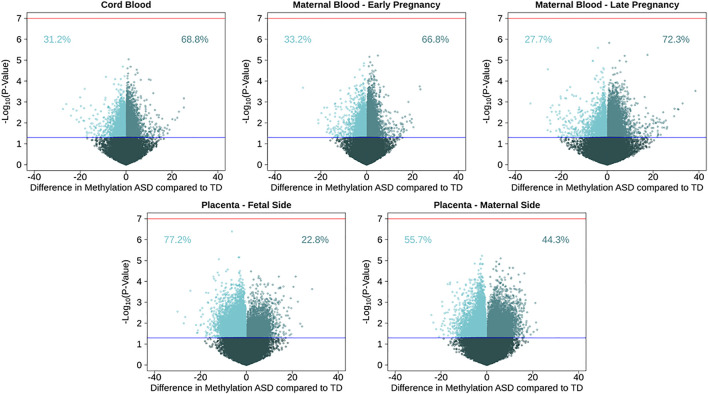
Volcano plots for single CpG site associations comparing ASD cases to a typical development reference group, showing percent methylation and –log_10_(*P*-values). Models were run in five tissues: maternal blood in early and late pregnancy, cord blood, and placental tissue on the fetal and maternal sides. Red line represents genome wide significance level of 10^–7^, and blue line represents nominal *p*-value of 0.05. Percentages show proportion of nominally significant CpG sites with positive or negative effect estimate.

After adjusting for multiple comparisons, no individual cord blood CpG sites reached genome-wide significance for association with ASD (Figure 1). In cord blood, we identified 215 single CpG sites associated with ASD diagnosis at a *P* < 0.001, and 17 reached *P*-value < 1 × 10^–4^ (Table 2). Specifically, we observed 1.12% higher methylation in the cord blood of ASD cases at cg17511416, a site on chromosome 9 that is related to the Coiled-Coil Domain Containing 171 (*CCDC171*) gene (Supplementary Figure 7). Out of 19,801 CpG sites with nominal association (*P*-value *<* 0.05), 68.8% were hypermethylated in cord blood.

**TABLE 2:** Cord blood DNA methylation loci associated (*P*-value < 1 × 10^–4^) with ASD diagnosis at 36-months, adjusting for surrogate variables.

**Probe ID**	**Gene symbol**	**Chr**	**Location**	**Mean percent methylation**	**Difference in percent methylation**	**Standard error**	***P*-value**
cg17511416	*CCDC171/C9orf93*	chr9	15553286	4.9	1.12	0.23	9.1 × 10^–6^
cg07921503	*CHST12*	chr7	2445843	8.3	–1.44	0.32	2.0 × 10^–5^
cg03421300	*SFN*	chr1	27189787	89.3	1.98	0.43	2.1 × 10^–5^
cg10621315	–	chr12	129276160	93.7	0.64	0.14	2.5 × 10^–5^
cg22918043	*C22orf41*	chr22	51001387	5.1	4.62	1.03	2.9 × 10^–5^
cg01071966	*CRHBP*	chr5	76248923	26.5	5.02	1.15	4.0 × 10^–5^
cg23957736	*FOXK2*	chr17	80530229	92.8	0.89	0.2	4.4 × 10^–5^
cg08333931	*SH3D20*	chr17	43507875	68.1	4.05	0.94	4.9 × 10^–5^
cg26357241	*STXBP3*	chr1	109289876	3.1	0.63	0.15	5.6 × 10^–5^
cg02387946	–	chr6	169092705	94.5	–0.64	0.15	5.8 × 10^–5^
cg18870850	*OGFR*	chr20	61439707	51.8	–8.78	2.07	6.3 × 10^–5^
cg04481212	*BDNF*	chr11	27740495	4	0.63	0.15	7.4 × 10^–5^
cg26526047	*KIAA1217*	chr10	23986387	89.9	-2.29	0.55	8.0 × 10^–5^
cg01421309	*ADPRHL1*	chr13	114084041	21.5	-4.77	1.14	8.1 × 10^–5^
cg14771419	–	chr7	142012988	45.1	9.94	2.38	8.4 × 10^–5^
cg24249791	–	chr7	142012611	61.1	6.39	1.54	8.8 × 10^–5^
cg05313263	*WDR25*	chr14	100906689	29	10.7	2.58	8.9 × 10^–5^

*Difference reflects ASD cases – typically developing controls.*

To test tissue specifcity we performed similar analyses in the other tissues. Consistent observations were also seen in maternal blood, where 66.8% of 20,454 nominally associated CpGs were hypermethylated in blood from early pregnancy, and 72.3% of 18,180 nominal CpGs were hypermethylated in late pregnancy. In early pregnancy, the most highly associated CpG site with ASD (*P*-value = 6 × 10^–6^) was cg23100720, annotated to the NADPH Oxidase 3 (*NOX3*) gene on chromosome 6, where maternal blood had 4.9% higher DNA methylation among those with a child with ASD. In late pregnancy, the most highly associated CpG site with ASD (*P*-value = 1 × 10^–6^) was cg05167232, annotated to the Heme Binding Protein 2 (*HEBP2*) gene on chromosome 6, where maternal blood had 0.87% higher DNA methylation among those with a child with ASD. Among nomially associated CpGs in placenta tissue, the opposite pattern of methylation direction was observed, where 22.9% of 23,621 nominally associated CpGs in fetal side tissue were hypermethylated, and 44.3% of 24,896 nominal CpGs were hypermethylated in maternal side placenta (Supplementary Table 6). On the fetal side of the placenta, the most highly associated CpG site with ASD (*P-*value = 4 × 10^–7^) was cg27202475, not annotated to any genes on chromosome 11, where placental tissue had 6.2% lower DNA methylation among those with ASD. On the maternal side of the placenta, the most highly associated CpG site with ASD (*P-*value = 6 × 10^–6^) was cg18908017, annotated to the Diacylglycerol Kinase Zeta (*DGKZ*) gene on chromosome 11, where placental tissue had 2.2% lower DNA methylation among those with ASD.

Effect estimates for the five tissues were most highly correlated between fetal and maternal side placenta (*r* = 0.35) and the maternal blood at two points in pregnancy (*r* = 0.32) (Supplementary Figure 8). The results for the primary model of ASD associations at all CpG sites in all five tissues (maternal blood early pregnancy, maternal blood late pregnancy, cord blood, fetal side placenta, and maternal side placenta) are included in Supplementary Table 7.

In sensitivity analyses, effect estimates from our primary models using a smaller number of surrogate variables were highly correlated with effect estimates generated from models using all surrogate variables. The Pearson correlation coefficients within tissue for the two types of surrogate variable models were 0.92 for cord blood, 0.89 for maternal early pregnancy blood, 0.85 for maternal late pregnancy blood, 0.77 for fetal side placenta, and 0.73 for maternal side placenta (all *P*-values for correlations < 2.2 × 10^–16^). Results from our sensitivity models adjusted with known covariates were also highly correlated with our primary surrogate variable models. Sensitivity models using known covariates had effect estimates that were highly correlated with surrogate variable models (cord blood *r* = 0.77, early pregnancy maternal blood *r* = 0.81, late pregnancy maternal blood *r* = 0.72, fetal placenta *r* = 0.70, maternal placenta *r* = 0.72) (Supplementary Figure 9). The full results at all sites are provided (Supplementary Table 8). Sensitivity models with DNA methylation on the *M*-value scale were also highly correlated with models on the beta-value scale (cord blood known covariate model *r* = 1.0).

### Testing DNA Methylation Sites for Enrichment in Autism Spectrum Disorder Genetic Loci

**FIGURE 2:**
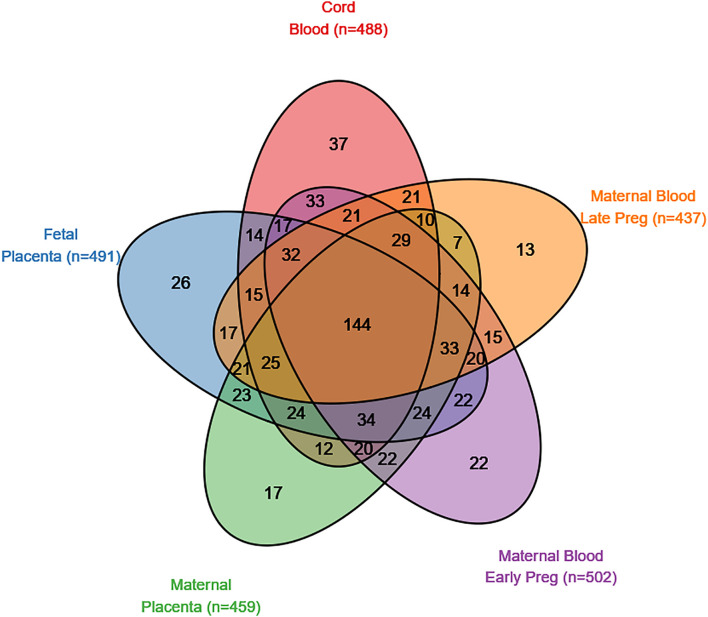
SFARI genes with a CpG site nominally associated (*p* < 0.05) with autism spectrum disorder in five tissues (maternal blood from early pregnancy, maternal blood from late pregnancy, cord blood, the fetal side of the placental tissue, and the maternal side of the placental tissue).

There were 839 SFARI genes with annotated CpGs in the analysis set. In cord blood, 488 (58.2% of SFARI genes represented by CpGs in analysis) SFARI genes had a CpG with *P* < 0.05, and 144 (29.5%) of these overlap between all five tissues (Figure 2). In non-SFARI genes, 7742 (42.6% of non-SFARI genes considered) had a CpG with *P* < 0.05 in cord blood, and 1221 (15.8%) overlapped with all other tissues (Supplementary Figure 10).

**FIGURE 3:**
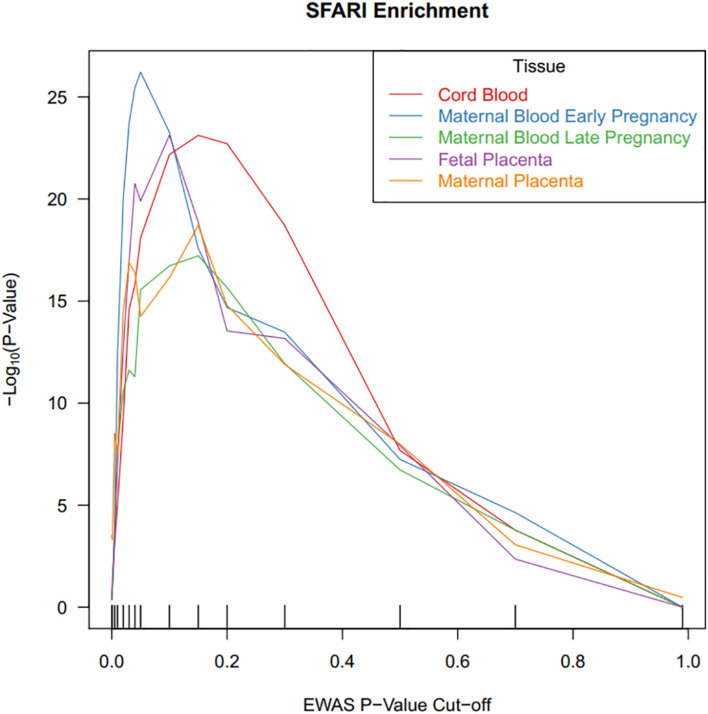
CpG sites associated with ASD status in cord blood are highly enriched for ASD risk genes. DNA methylation sites were tested for association with ASD status, and the *x*-axis illustrates *P*-value criteria selecting DNA methylation sites based on these association tests. The DNA methylation sites were mapped to genes and these genes were tested for enrichment in ASD risk genes. The *y*-axis demonstrates the –log_10_*(P*-value) for these enrichment tests. Line colors are based on the tissue measured for DNA methylation.

We tested the ASD-associated cord blood DNA methylation sites at multiple significance cutoffs for enrichment in SFARI annotated ASD genes (Figure 3). Among CpG sites associated with ASD status in cord blood at a nominal *P*-value of 0.05, we observed a chi-square test enrichment *P*-value = 7.9 × 10^–19^. At this level, among 8,230 genes associated with ASD in cord blood, we observed 488 overlapping with 839 total SFARI genes, compared to the expected overlap of 363 genes. Similar levels of enrichment were observed in the other tissues among CpG sites nominally associated at a *P-*value of 0.05. In early pregnancy maternal blood, we observed an enrichment *P* value of 6.1 × 10^–27^, corresponding to an overlap of 502 genes compared to the expected of 350. In late pregnancy maternal blood, we observed an enrichment *P*-value of 2.8 × 10^–16^, with 437 genes overlapping compared to 322 expected. In fetal side placenta 491 genes overlapped compared to 360 expected, giving an enrichment *P-*value of 1.3 × 10^–20^. Finally, in maternal side placenta there was an enrichment *P*-value of 5.6 × 10^–15^ with 459 genes overlapped compared to an expected of 349.

Autism spectrum disorder -associated DNA methylation sites were not associated with any of our negative control permutation analyses using a random set of genes (Supplementary Figure 11). Enrichment of SFARI genes in cord blood CpGs associated with ASD was minimal when restricting to meQTL targets, compared to enrichment when considering all CpG data (Supplementary Figure 12).

### Gene Ontologies Associated With Autism Spectrum Disorder Status

Among the individual CpG sites most associated with ASD in each tissue, we tested for enrichment of biological processes. There were 192 unique annotated entrez gene IDs mapped to gene ontology biological processes. The biological processes with lowest rank sum across the five tissues were cell adhesion (rank sum of 4466), histone H3-K36 demethylation (rank sum of 4485), and regulation of nucleotide biosynthetic process (rank sum 4594), though none of these processes had at least *p* < 0.05 in all five tissues (Supplementary Table 9).

### Sensitivity Analysis Comparing DNA Methylation for Non-typically Developing Relative to Typically Developing

In our study sample, 42.9% of participants were non-typically developing, but not diagnosed with ASD. In cord blood, comparing non-typically developing to typically developing 66.5% of 16,977 nominally associated CpGs (*P*-value *<* 0.05) had higher DNA methylation in the non-typically developing group (Supplementary Figure 13). Effect estimates for the non-typically developing vs. typically developing model and the ASD vs. typically developing model had Pearson correlation of 0.47 (Supplementary Figure 14). Non-typically developing associated CpGs were also enriched in SFARI genes (Supplementary Figure 15). Single CpG results for associations with non-typically developing status for all five tissues (maternal blood early pregnancy, maternal blood late pregnancy, cord blood, fetal side placenta, and maternal side placenta) are included in Supplementary Table 10. Additionally, single CpG results for all five tissues using known covariate models are included in Supplementary Table 11.

## Discussion

In a prospective pregnancy cohort, we observed ASD associated DNA methylation sites measured in cord blood, maternal blood, and placenta tissue were enriched in previously identified ASD-associated genes. No individual CpG sites reached genome-wide significance when comparing the ASD group and typically developing group. Among cord blood CpG sites reaching *P*-value < 0.001, genes were enriched for cell adhesion pathways. Our findings of autism genetic loci enrichment in DNA methylation from five perinatal tissue types support that for a subset of genomic sites, non-brain tissues in the perinatal period may still be informative for ASD.

In the EARLI enriched-risk pregnancy cohort study sample, 21.8% of at-risk siblings were diagnosed with ASD by age 3 years. This is consistent with previous literature reports on the prevalence of ASD among siblings with ASD (Constantino et al., 2010; Ozonoff et al., 2011). We also observed 75.9% of ASD cases born in the study were male, which again is consistent with well-documented sex-differences in ASD (Werling and Geschwind, 2013). Study site specific differences in ASD prevalence were driven by differences in infant sex. Though previous studies have identified ASD risk associated with advanced parental age (Croen et al., 2007; Sandin et al., 2012), race/ethnicity (Christensen et al., 2016), and parental education (Durkin et al., 2010), we did not observe these relationships in our small, at-risk study sample.

Pathology for autism is likely centrally located in the brain, implying that brain-based epigenetic analysis is most appropriate. Indeed, DNA methylation differences with ASD status have been observed in post-mortem brain tissue (Nagarajan et al., 2006; Ladd-Acosta et al., 2014). However, epidemiological studies of ASD in brain tissue are plagued with challenges such as small sample size, limited replication, and timing of collection after disease manifestation and aging (Bakulski et al., 2016b). Perinatal tissue studies of ASD may lack the same target tissue relevance of brain studies, but they have advantages due to larger potential sample sizes and timing prior to disease onset (Bakulski et al., 2016b). Peripheral tissues have been interrogated for ASD case/control DNA methylation differences, with consistent signals to brain reports (Andrews et al., 2018). Common DNA methylation changes with ASD have been observed across brain and blood at methylation quantitative trait loci, where polymorphic genotypes control DNA methylation levels (Andrews et al., 2017). Similarly, polygenic burden for ASD was associated with neonatal blood spot DNA methylation (Hannon et al., 2018). Those observations combined with the current study emphasize the potential for cross-tissue molecular ASD studies to overcome logistical and design challenges inherent to brain, or any single tissue, studies.

An advantage of using perinatal tissues in ASD epidemiology studies is that they are less likely to represent a consequence of pathogenesis and more likely to represent an intermediate step in the disease process. Similar to previous genome-wide DNA methylation measures in human placenta (Schroeder et al., 2013), we observed higher relative abundance of partially methylated domains (PMDs) compared to peripheral and cord blood tissues. Placental regulation of nutrients, waste, and oxygen (Tarrade et al., 2015), as well as hormone generation that controls fetal growth and survival (Ilekis et al., 2016), have spurred expanded interest in neuroplacentology (Roescher et al., 2014). In model systems, gamete epigenetic modifications inherited from parents can influence offspring development (Kaneshiro et al., 2019). Paternal semen samples are another promising perinatal tissue for ASD DNA methylation studies (Feinberg et al., 2015). Our study is the first to measure maternal peripheral blood DNA methylation during pregnancy for association with ASD in the offspring. DNA methylation from maternal, paternal, and placental tissues from the pre-conception and pregnancy periods have been associated with ASD status in children.

Our study sample of 133 cord bloods with paired ASD diagnosis at age 3 years is underpowered for single site epigenome-wide association studies (Tsai and Bell, 2015), and no single CpG achieved genome-wide statistical significance in our sample. With larger sample sizes, it is likely that true associations will emerge, but are currently among this set of nominally significant CpGs and cannot be distinguished from “noise.” Future work in consortia, such as the Pregnancy And Childhood Epigenetics (PACE) consortium (Felix et al., 2018) may be able to build upon these promising findings in the EARLI study and meta-analyze across studies for CpG-level interrogation. In our study, the set of nominally associated CpG sites was enriched for genes previously associated with ASD, indexed in the SFARI curated database. This suggests, at a common set of genes, genetic or epigenetic dysregulation is associated with ASD development. Converging evidence is supporting the importance of these genes. DNA methylation is correlated over smaller genomic distances than genetic linkage disequilibrium blocks (Sun and Sun, 2019) and can be used to fine tune genome-wide association study locations for functional study (Andrews et al., 2017). These ASD associated genes in common from genetic and epigenetic studies should be prioritized for biomarker testing in an independent sample, biologic interrogation, or mechanistic study.

Aggregate DNA methylation across sites by subset of the regulatory genome had suggestive associations with ASD status in the fetal placenta tissue, showing modest hypomethylation. However, effect sizes were small and are not significant when accounting for multiple testing. Small magnitude effect sizes have been highlighted as expected and important in children’s health studies with other health outcomes (Breton et al., 2017). In fetal placenta the strongest relationships with global methylation were seen in the shelf and open sea regions. Since open sea regions are not necessarily linked to specific genes, DNA methylation differences with ASD suggest alternate, non-genic mechanisms of ASD biology. Indeed, genes involved in ASD, such as Chromodomain Helicase DNA Binding Protein 8 (*CHD8*), implicate chromatin modeling more generally (De Rubeis et al., 2014). Hypomethylation was observed in the open sea regions of ASD cases relative to controls in multiple perinatal tissues.

## Conclusion

We tested for genome-wide DNA methylation differences by ASD status in cord blood and in maternal blood at two time points during pregnancy and from two sides of the placenta. This is the most comprehensive, multiple perinatal tissue investigation of epigenetics in ASD to date. DNA methylation sites that were nominally associated with ASD were highly enriched for ASD risk genes in all tissues. Importantly, findings from this enriched-risk pregnancy cohort should be tested for replication and for generalizability to population-based samples. Perinatal tissues may be effective biosamples for investigating ASD prior to or concurrent with developmental pathogenesis. Converging evidence from genetic and epigenetic studies implicate dysregulation of common genes in ASD.

## Data Availability Statement

The datasets presented in this study can be found in online repositories. The names of the repository/repositories and accession number(s) can be found below: https://nda.nih.gov/edit_collection.html?id=1600, 1600.

## Ethics Statement

The studies involving human participants were reviewed and approved by Human Subjects Institutional Review Boards from each of the four study sites (Johns Hopkins University, Drexel University, University of California, Davis, and Kaiser Permanente Northern California). Secondary analysis of data was reviewed and approved by University of Michigan Institutional Review Board. Written informed consent to participate in this study was provided by the participants’ legal guardian/next of kin.

## Author Contributions

CN, LC, IH-P, and MF developed the cohort. JF processed the biologic samples. RL and SL provided the clinical diagnoses and interpretation. MF, KB, and AF conceptualized and designed the project. MF and AF funded and supervised the project. JD, KB, and MA performed the analyses and code review. KB and JD drafted the manuscript. CL-A, HV, MF, and JF provided the manuscript revisions and editing. All authors reviewed the results and approved the submitted version of the manuscript.

## Conflict of Interest

The authors declare that the research was conducted in the absence of any commercial or financial relationships that could be construed as a potential conflict of interest.

## Publisher’s Note

All claims expressed in this article are solely those of the authors and do not necessarily represent those of their affiliated organizations, or those of the publisher, the editors and the reviewers. Any product that may be evaluated in this article, or claim that may be made by its manufacturer, is not guaranteed or endorsed by the publisher.

## Footnotes

1
www.sfari.org

2
https://github.com/bakulskilab

## Abbreviations

ASD: autism spectrum disorders
CpG: cytosine guanine dinucleotide
SFARI: Simons Foundation Autism Research Initiative.
