# Supplementary material for: Autism-Associated DNA Methylation at Birth From Multiple Tissues Is Enriched for Autism Genes in the Early Autism Risk Longitudinal Investigation
Source: Front Mol Neurosci. 2021 Nov 25;14:775390. doi: 10.3389/fnmol.2021.775390 (PMC8655859; doi:10.3389/fnmol.2021.775390)
Supplement: Supplementary file 1 [file Data_Sheet_1.DOCX]

Autism-associated DNA methylation at birth from multiple tissues is enriched for autism genes in the Early Autism Risk Longitudinal Investigation (EARLI)

Supplementary Information. Contains 15 supplemental figures and 5 supplemental tables. Additional supplemental tables are available as excel spreadsheets.


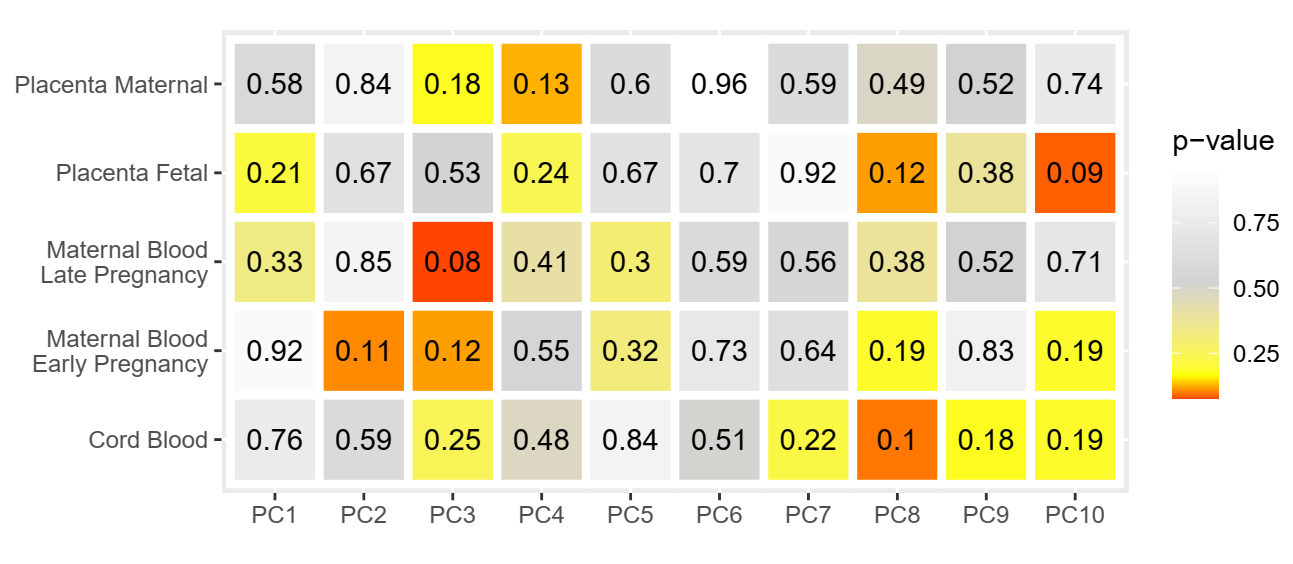
**Supplementary Figure 1**. Associations between principal components of DNA methylation data (x-axis) and study site in each tissue type (y-axis). Numbers represent p-value for ANOVA test for association of principal component and study site as a categorical variable.


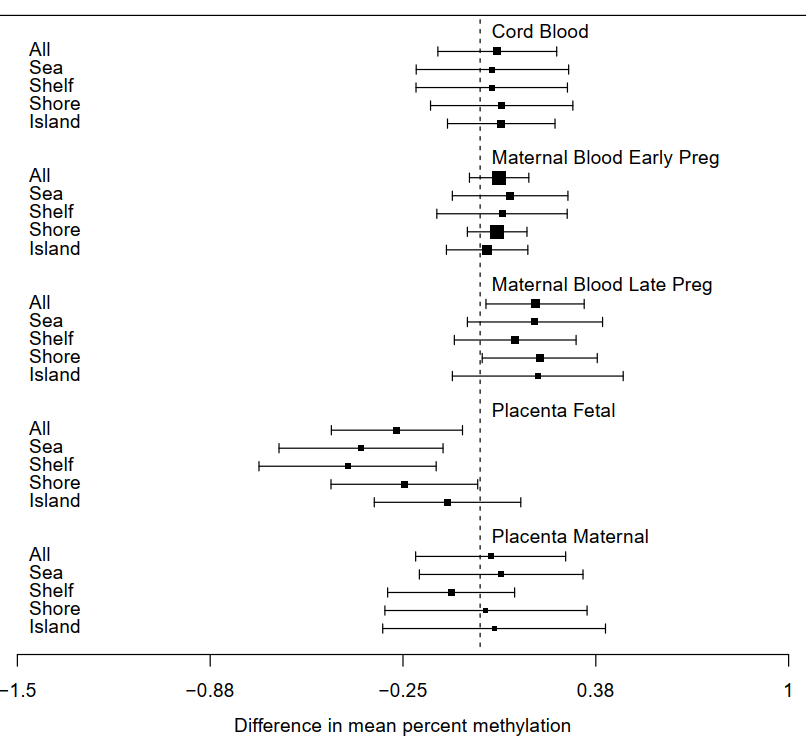


**Supplementary Figure 2**. Difference in global DNA methylation, calculated as mean methylation across all probes and by genomic regions, adjusted for surrogate variables.


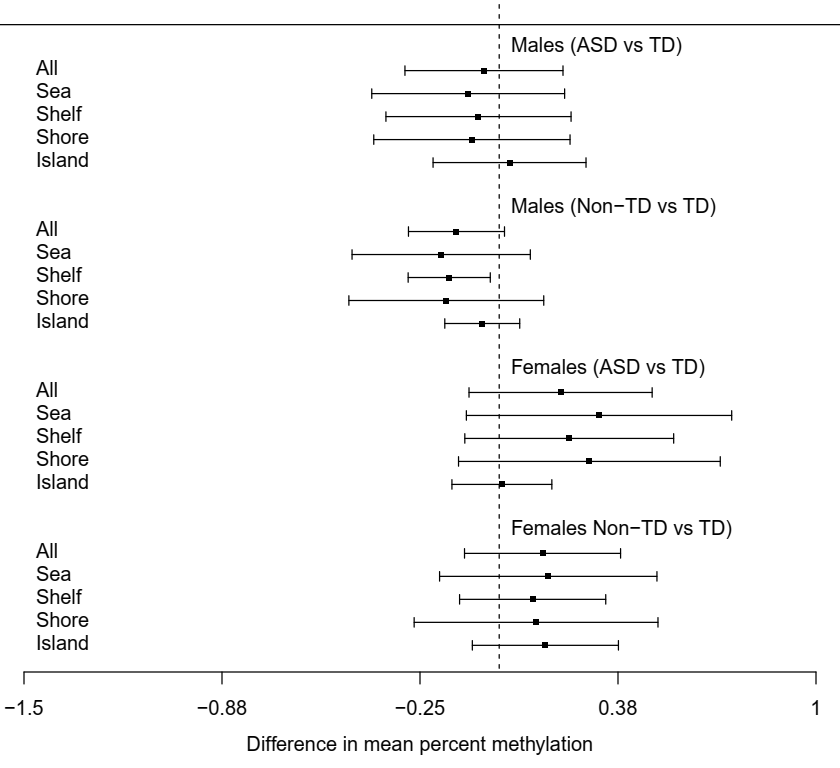
**Supplementary Figure 3**. Difference in global DNA methylation stratified by sex, calculated as mean methylation across all probes and by genomic regions, adjusted for surrogate variables.

| A.  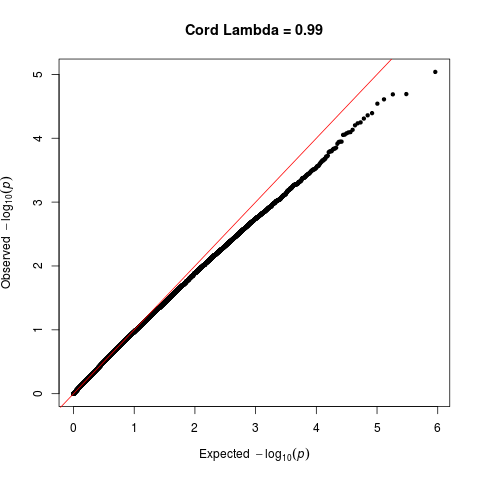 | B.  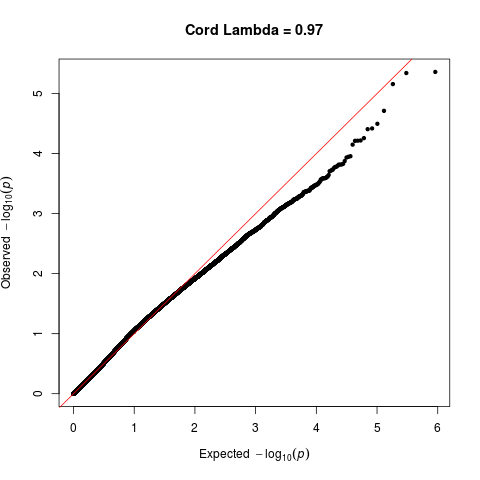 |
| --- | --- |

**Supplementary Figure 4**. Expected versus observed P-value distribution for the association between ASD and DNA methylation. A. Adjusted for the first three surrogate variables only (no measured covariates)

B. Adjusted for measured covariates (batch + baby’s sex + PC1 for ancestry + maternal age + estimated percent granulocytes + estimated percent nucleated red blood cells).


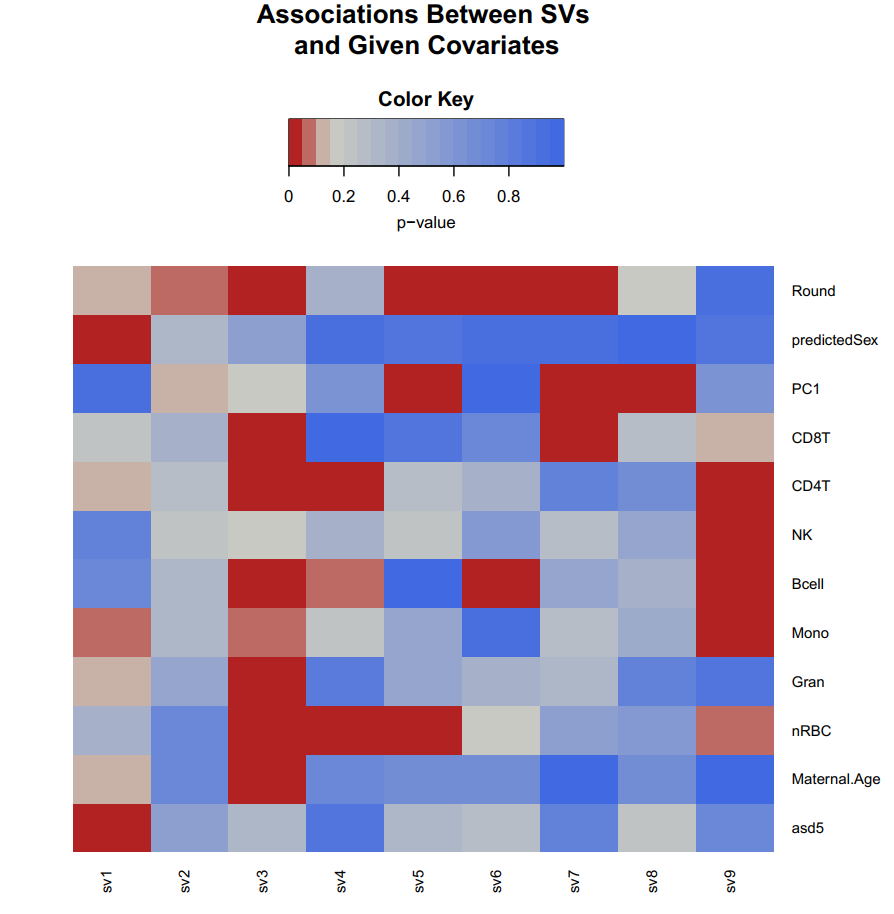


**Supplementary Figure 5**. Statistical significance for the correlation between measured covariates and surrogate variables (SV’s).

| A.  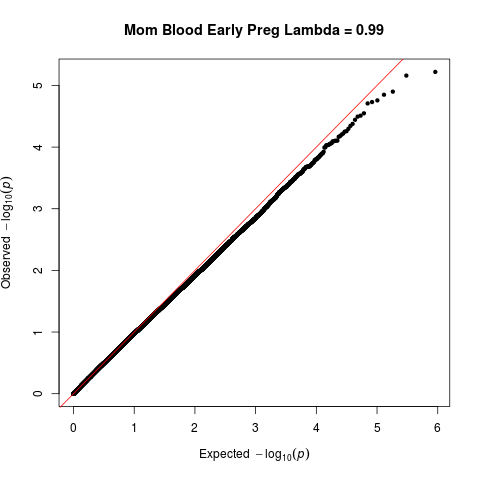 | B.  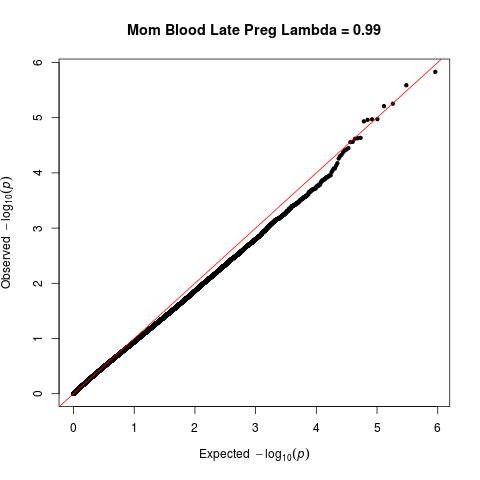 |
| --- | --- |
| C.  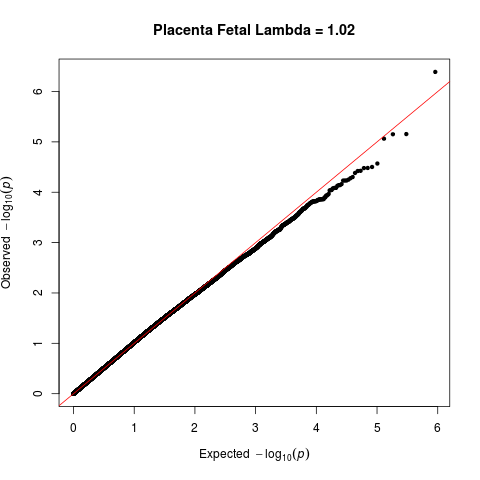 | D.  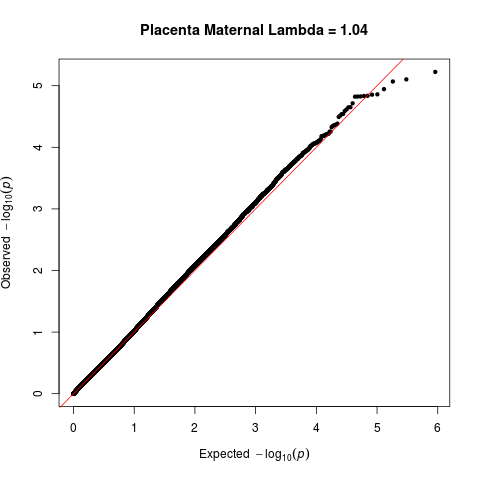 |

**Supplementary Figure 6**. Expected versus observed P-value distribution for the association between ASD and DNA methylation in maternal blood in early (A) and late (B) pregnancy, and placental tissue on fetal (C) and maternal (D) side, adjusted for surrogate variables.


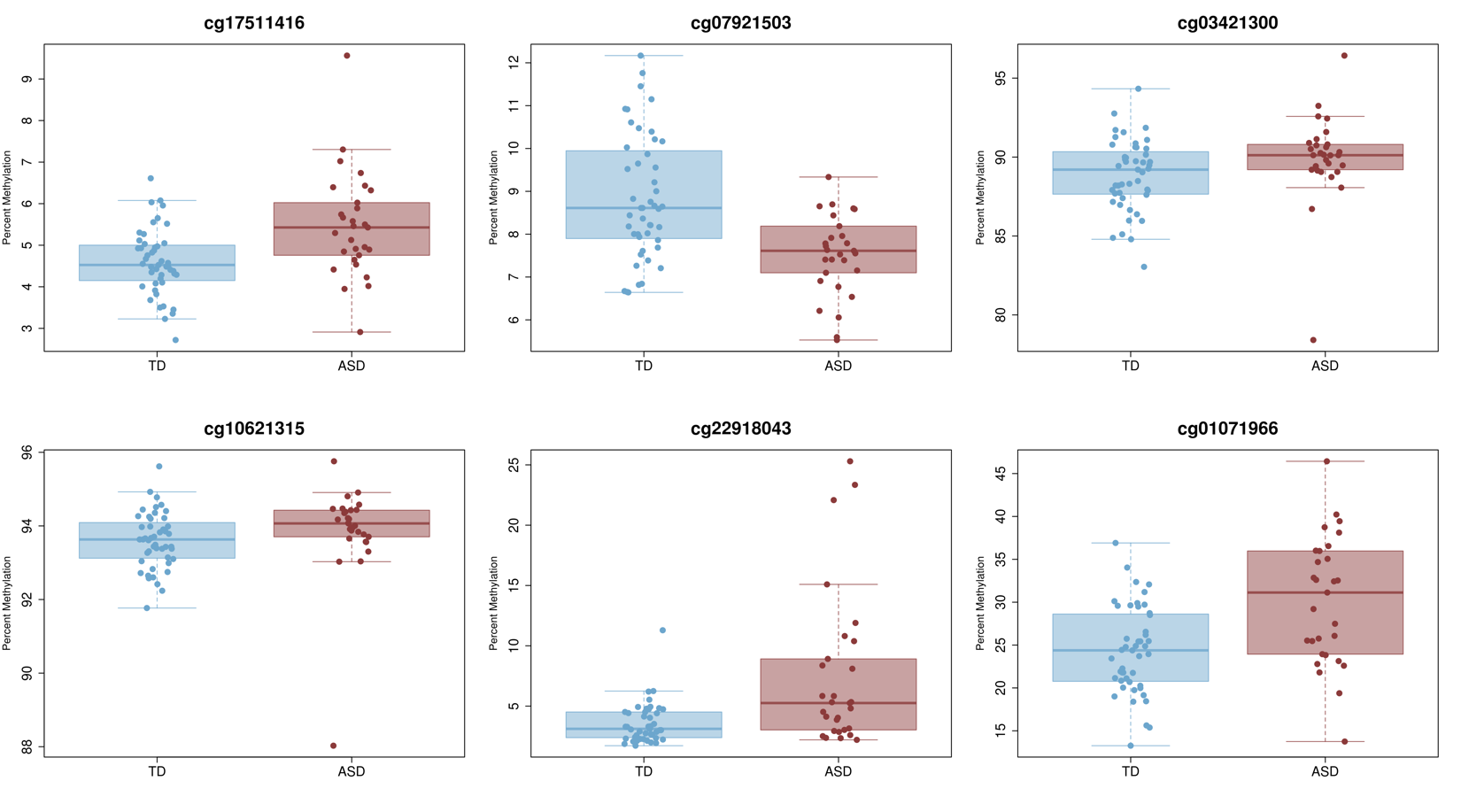


A.

B.

C.

D.

E.

F.

**Supplementary Figure 7**. Cord blood DNA methylation sites associated with ASD status as 36-months. Six sites highest ranked statistically. Red is ASD cases. Blue is typically developing. A. cg17511416 B. cg07921503 C. cg03421300 D. cg10621315 E. cg22918043 F. cg01071966


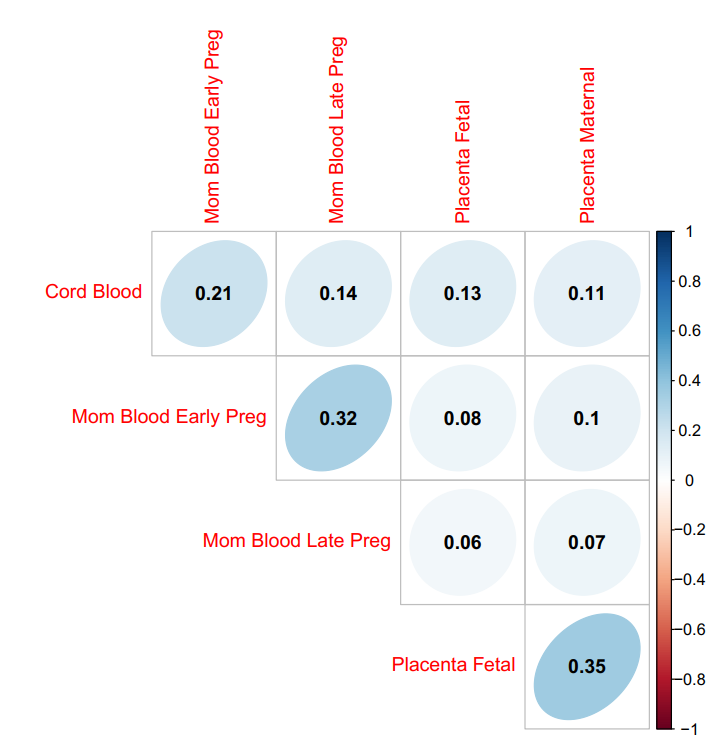


**Supplementary Figure 8**. Correlation matrix for regression effect estimates for DNA methylation from ASD compared to typically developing. Regression models used surrogate variables across five tissues (cord blood, fetal side of the placenta, maternal side of the placenta, maternal blood from early pregnancy, maternal blood from late pregnancy).

| **A.**  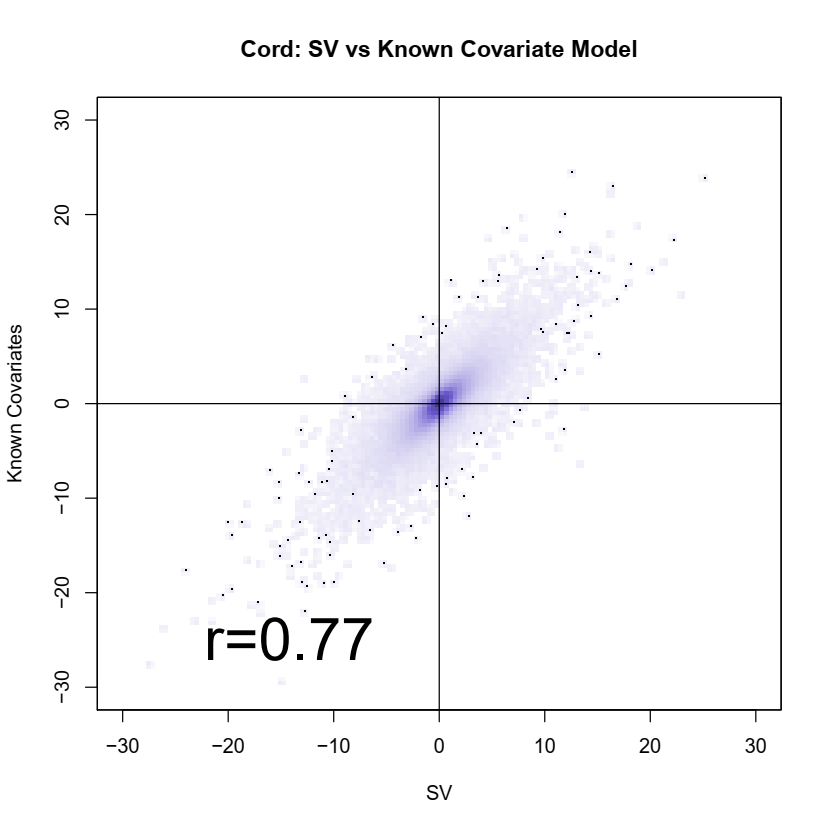 | | **B.**  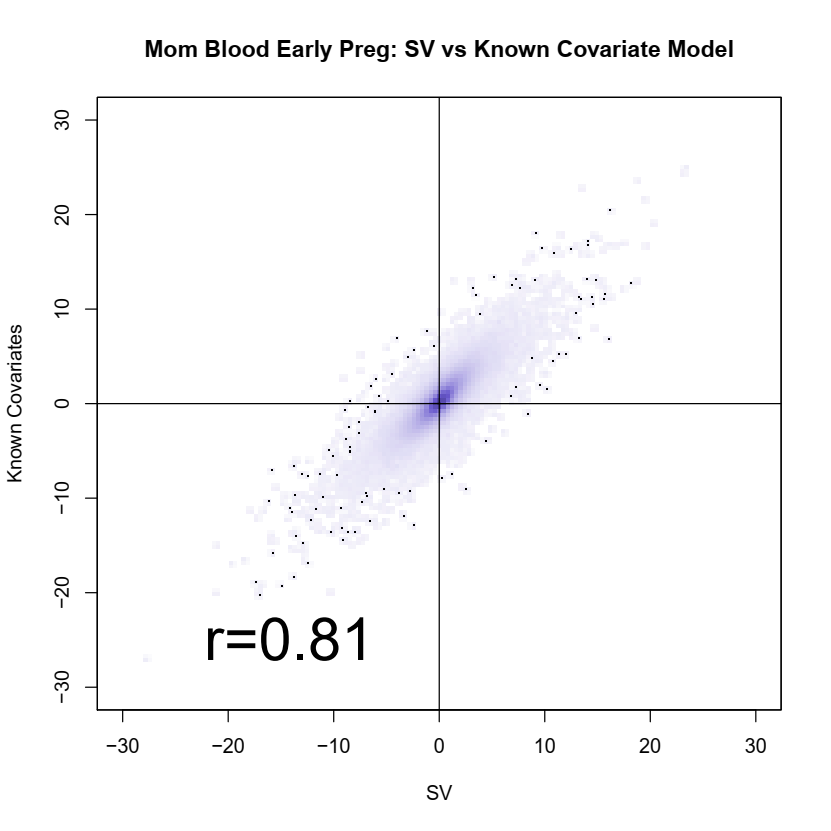 | |
| --- | --- | --- | --- |
| **C.**  . 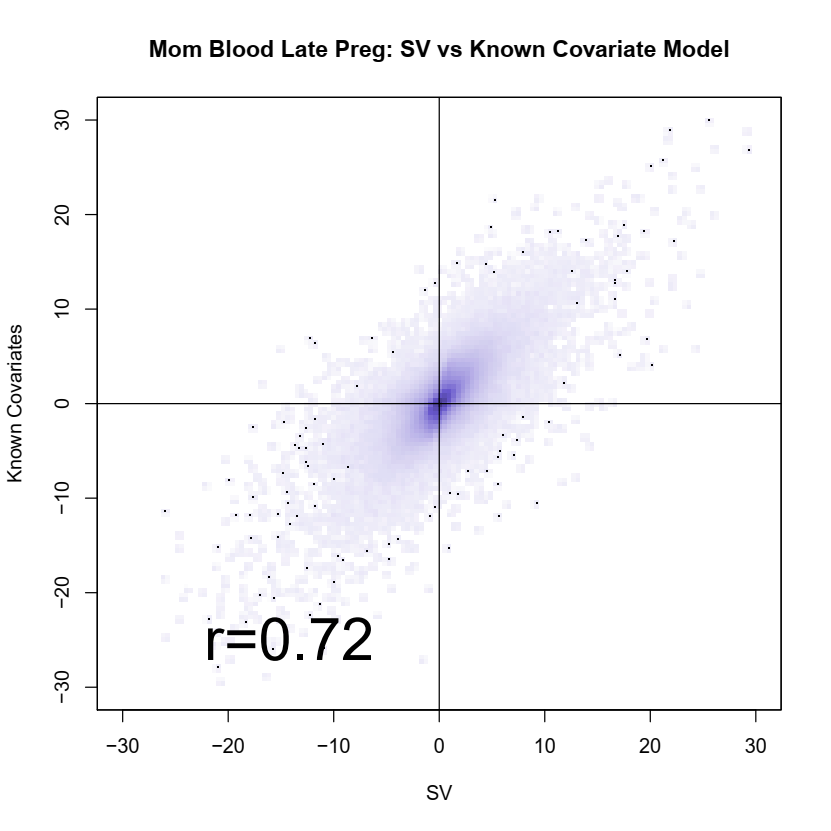 | | **D.**  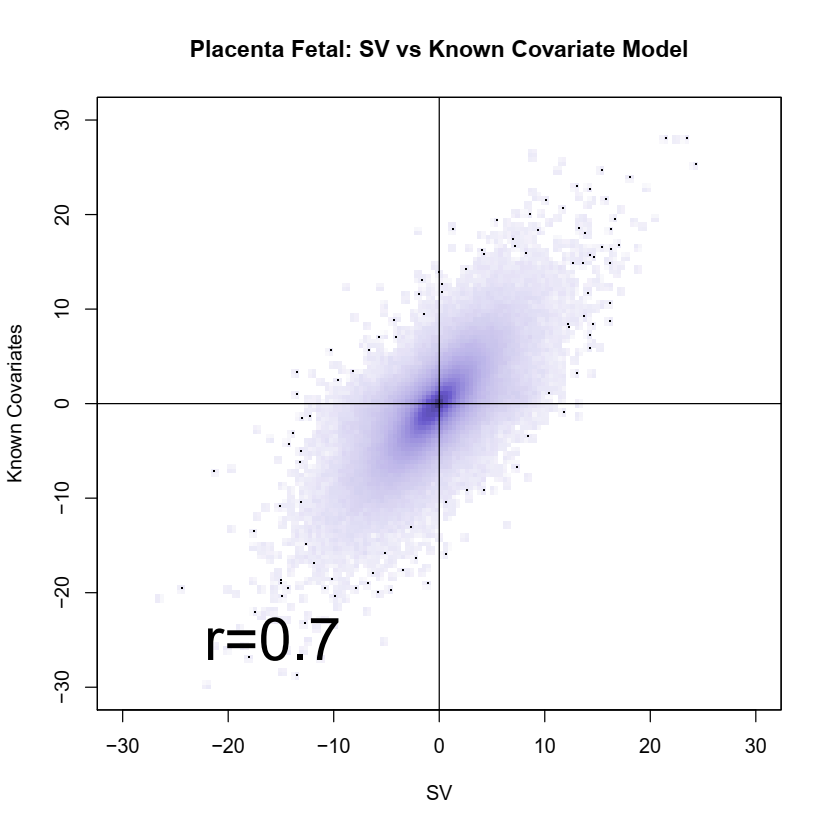 | |
|  | **E.**  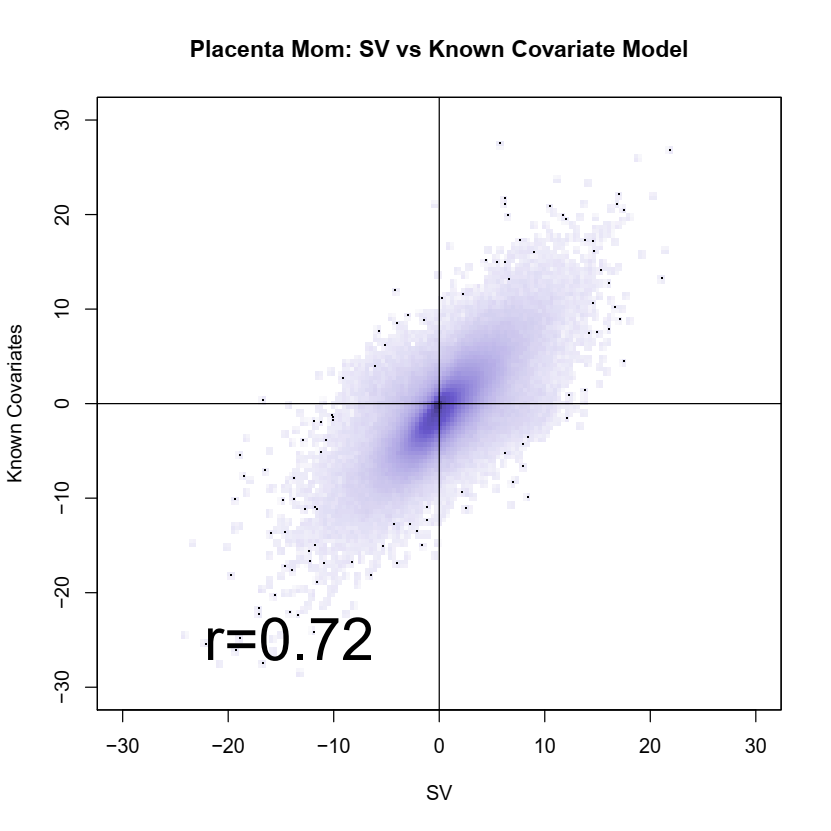 | |  |

**Supplementary Figure 9**. Correlation of effect estimates from models using surrogate variables (x-axis) and models using known covariates (y-axis) in A. cord blood, B. early pregnancy maternal blood, C. late pregnancy maternal blood, D. fetal side placenta, and E. maternal side placenta.


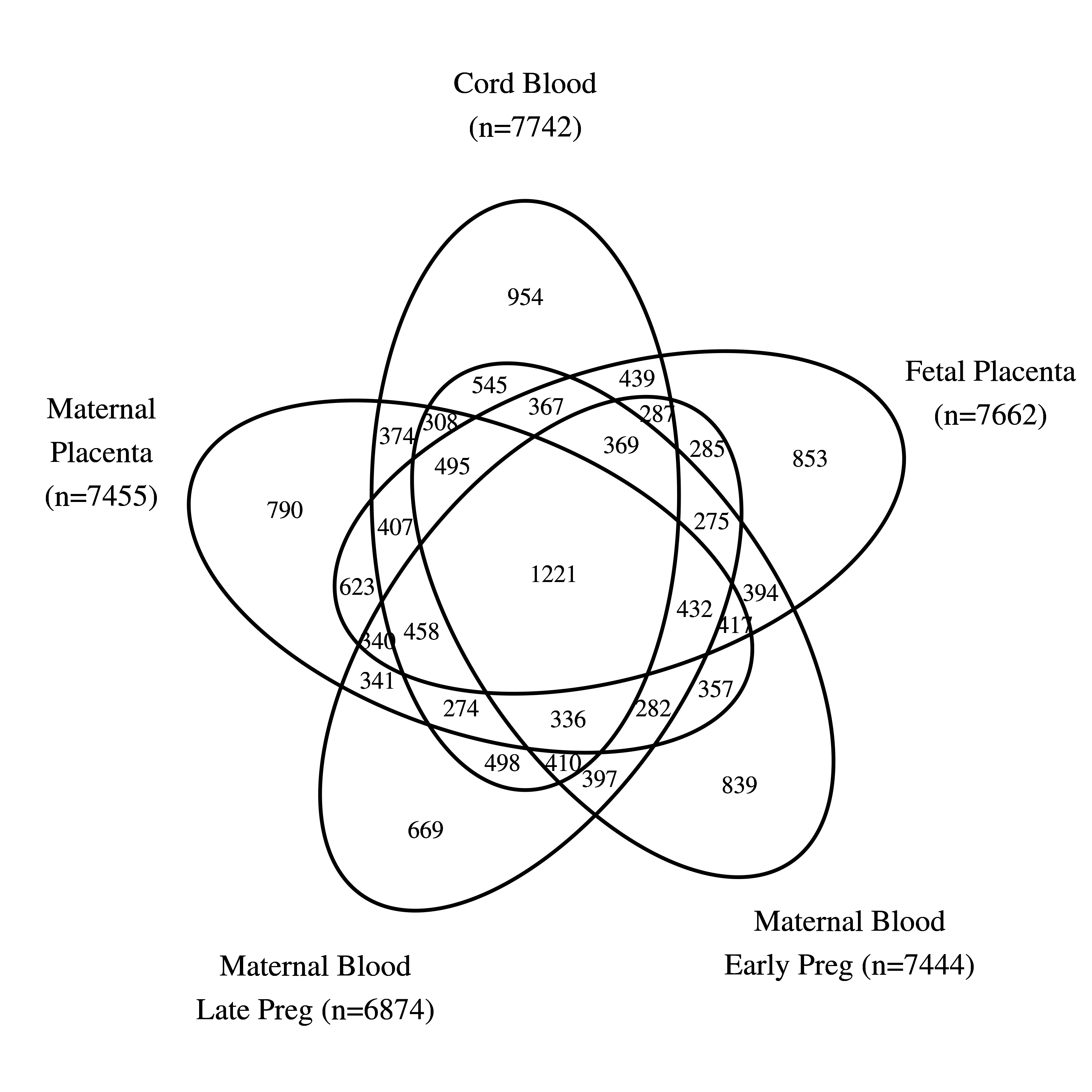


**Supplementary Figure 10**. Non-SFARI genes with a CpG site nominally associated (p<0.05) with ASD in 5 tissues.


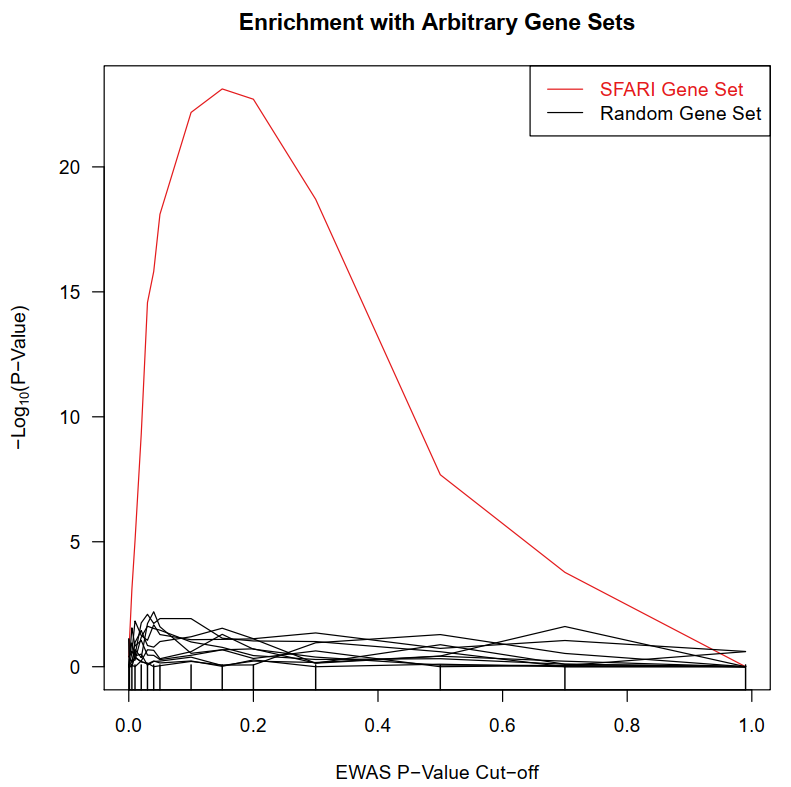


**Supplementary Figure 11**. Enrichment of SFARI gene set (red) in cord blood ASD associated CpG sites and enrichment of random gene sets of equal size (black).


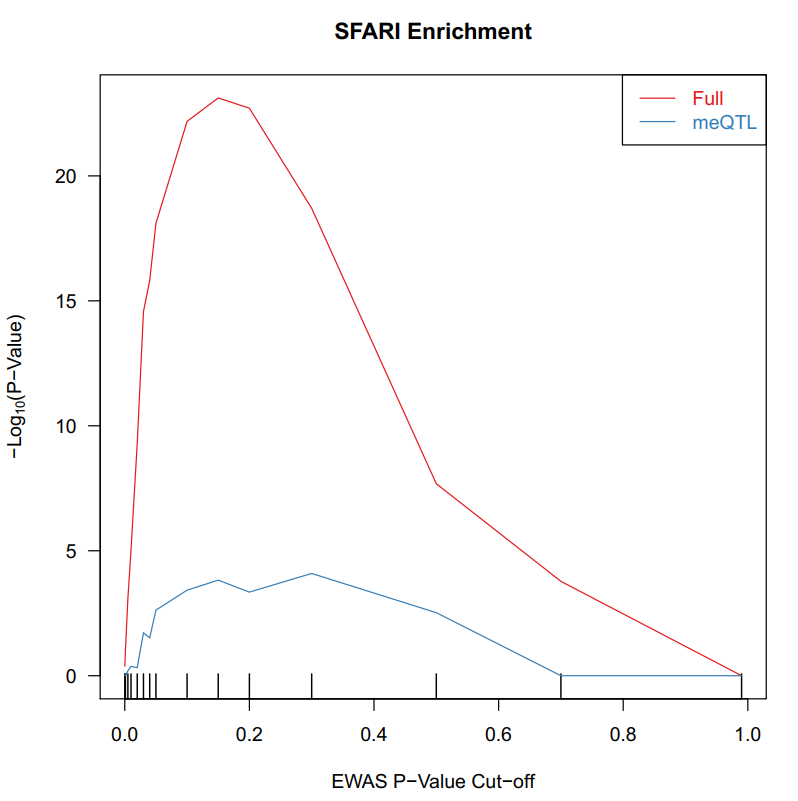


**Supplementary Figure 12**. Enrichment of SFARI genes in cord blood when (red) considering all CpGs, and when (blue) only considering meQTL targets.


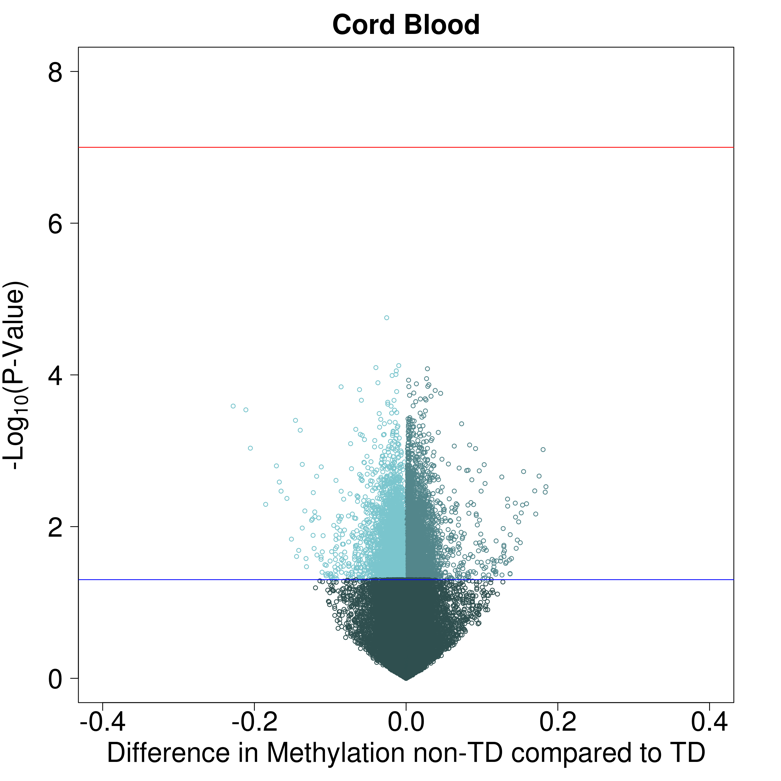


**Supplementary Figure 13**. Volcano plot for model comparing non-TD to TD, using two surrogate variables. Of the CpG’s with *P*>0.05, 66.5% had effect estimate > 0.


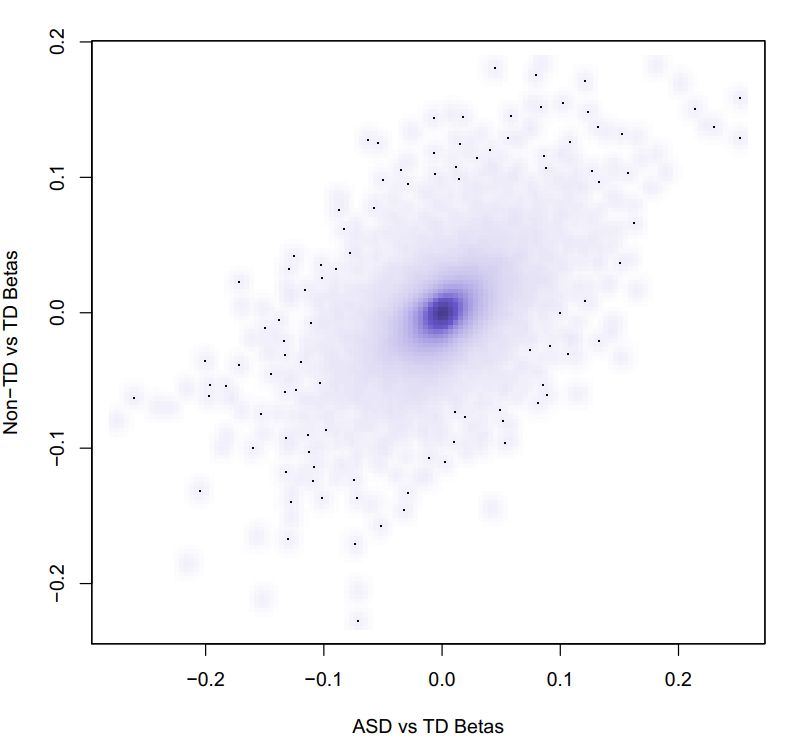


**Supplementary Figure 14**. In cord blood, effect estimates from models comparing ASD vs TD plotted against effect estimates from models comparing non-TD to TD groups.


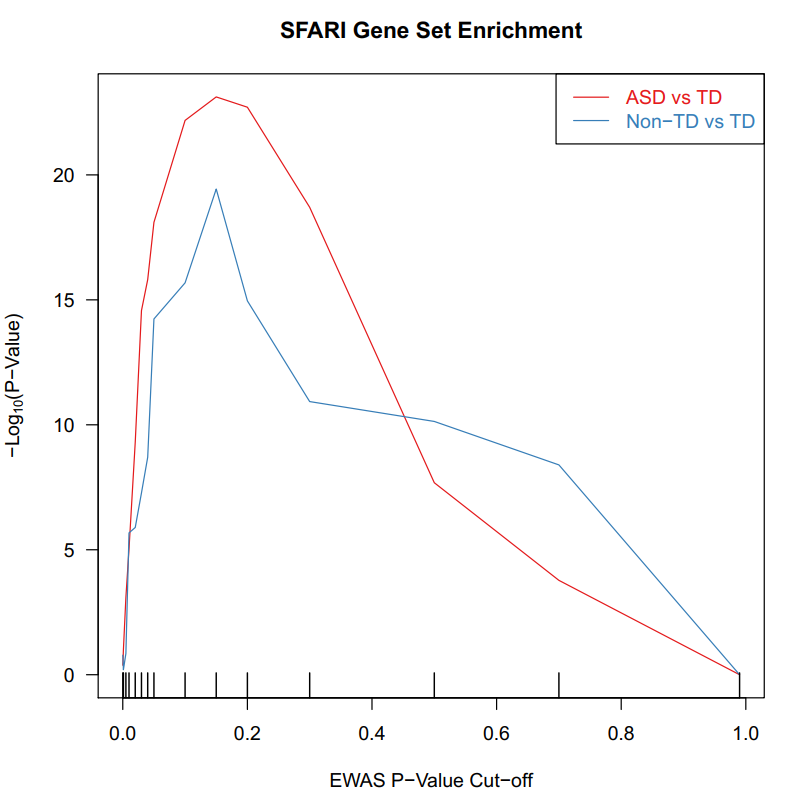


**Supplementary Figure 15.** Enrichment of SFARI autism risk genes in CpG sites associated with ASD vs TD status, and in CpG sites associated with non-TD vs TD status.

**Supplementary Table 1. Early pregnancy maternal blood study sample descriptive statistics by Baby Siblings Research Consortium algorithm typically developing, non-typically developing, and Autism Spectrum Disorder (ASD) categorization. Mean (SD) for continuous covariates and N(%) for categorical covariates.**

| **Covariate** | **Typically developing**  **N=55** | **Non-typically developing**  **N=60** | **ASD**  **N=28** | **P** |
| --- | --- | --- | --- | --- |
| **Sibling Sex** |  |  |  |  |
| Female | 30 (54.5%) | 32 (53.3%) | 6 (21.4%) | 0.008 |
| Male | 25 (45.5%) | 28 (46.7%) | 22 (78.6%) |  |
| **Maternal Age** |  |  |  |  |
| Continuous | 34.9 (5.01) | 33.2 (4.88) | 34.7 (4.28) | 0.131 |
| **Paternal Age** |  |  |  |  |
| Continuous | 36.3 (6.59) | 35.3 (5.82) | 35.4 (6.45) | 0.637 |
| Missing |  | 1 (1.67%) |  |  |
| **Race/Ethnicity** |  |  |  |  |
| Non-Hispanic White | 36 (65.5%) | 27 (45.0%) | 14 (50.0%) | 0.088 |
| Non-Hispanic Black | 2 (3.6%) | 8 (13.3%) | 3 (10.7%) |  |
| Hispanic/Latino | 4 (7.3%) | 12 (20.0%) | 7 (25.0%) |  |
| Other | 8 (14.5%) | 10 (16.7%) | 3 (10.7%) |  |
| Missing | 5 (9.09%) | 3 (5.0%) | 1 (3.6%) |  |
| **Maternal Education** |  |  |  |  |
| High school or Less | 5 (9.09%) | 7 (11.7%) | 7 (25.0%) | 0.062 |
| Some college | 13 (23.6%) | 20 (33.3%) | 9 (32.1%) |  |
| Bachelors Degree | 15 (27.3%) | 22 (36.7%) | 5 (17.9%) |  |
| Graduate Degree | 22 (40.0%) | 11 (18.3%) | 6 (21.4%) |  |
| Missing |  |  | 1 (3.6%) |  |
| **Household Income** |  |  |  |  |
| <$50,000 | 10 (18.2%) | 20 (33.3%) | 8 (28.6%) | 0.372 |
| $50,000 - $99,999 | 18 (32.7%) | 18 (30.0%) | 10 (35.7%) |  |
| > $100,000 | 26 (47.3%) | 21 (35.0%) | 9 (32.1%) |  |
| Missing | 1 (1.8%) | 1 (1.7%) | 1 (3.6%) |  |
| **Batch** |  |  |  |  |
| 1 | 3 (5.45%) | 1 (1.67%) | 3 (10.7%) | 0.116 |
| 2 | 52 (94.5%) | 59 (98.3%) | 25 (89.3%) |  |
| **Study Site** |  |  |  |  |
| Drexel | 22 (40.0%) | 9 (15.0%) | 5 (17.9%) | 0.003 |
| Johns Hopkins | 5 (9.09%) | 22 (36.7%) | 5 (17.9%) |  |
| Kaiser | 17 (30.9%) | 18 (30.0%) | 9 (32.1%) |  |
| UC Davis | 11 (20.0%) | 11 (18.3%) | 9 (32.1%) |  |
| **Cell Type Percent** |  |  |  |  |
| Granulocyte | 73.1 (7.11) | 71.6 (7.39) | 71.3 (6.38) | 0.422 |
| CD8+ Tcell | 5.44 (2.51) | 6.07 (2.78) | 5.87 (3.33) | 0.479 |
| CD4+ Tcell | 8.26 (3.83) | 8.73 (4.20) | 9.37 (4.61) | 0.514 |
| NK Cell | 2.27 (2.36) | 1.89 (2.15) | 2.72 (2.88) | 0.314 |
| Bcell | 3.15 (1.76) | 3.81 (1.79) | 3.55 (1.84) | 0.149 |
| Monocyte | 7.04 (2.06) | 7.25 (2.36) | 6.56 (3.44) | 0.481 |

**Supplementary Table 2. Late pregnancy maternal blood study sample descriptive statistics by Baby Siblings Research Consortium algorithm typically developing, non-typically developing, and Autism Spectrum Disorder (ASD) categorization. Mean (SD) for continuous covariates and N(%) for categorical covariates.**

| **Covariate** | **Typically developing**  **N=27** | **Non-typically developing**  **N=36** | **ASD**  **N=15** | **P** |
| --- | --- | --- | --- | --- |
| **Sibling Sex** |  |  |  |  |
| Female | 15 (55.6%) | 16 (44.4%) | 3 (20.0%) | 0.008 |
| Male | 12 (44.4%) | 20 (55.6%) | 12 (80.0%) |  |
| **Maternal Age** |  |  |  |  |
| Continuous | 34.0 (4.15) | 33.4 (4.37) | 33.8 (2.81) | 0.83 |
| **Paternal Age** |  |  |  |  |
| Continuous | 35.4 (6.09) | 36.1 (6.06) | 36.2 (5.09) | 0.88 |
| **Race/Ethnicity** |  |  |  |  |
| Non-Hispanic White | 17 (62.9%) | 13 (36.1%) | 7 (46.7%) | 0.084 |
| Non-Hispanic Black | 0 (0.0%) | 3 (8.3%) | 3 (20.0%) |  |
| Hispanic/Latino | 2 (7.4%) | 8 (22.2%) | 2 (13.3%) |  |
| Other | 4 (14.8%) | 10 (27.8%) | 2 (13.3%) |  |
| Missing | 4 (14.8%) | 2 (5.6%) | 1 (6.7%) |  |
| **Maternal Education** |  |  |  |  |
| High school or Less | 2 (7.41%) | 4 (11.1%) | 2 (13.3%) | 0.54 |
| Some college | 7 (25.9%) | 14 (38.9%) | 4 (26.7%) |  |
| Bachelors Degree | 7 (25.9%) | 12 (33.3%) | 4 (26.7%) |  |
| Graduate Degree | 11 (40.7%) | 6 (16.7%) | 4 (26.7%) |  |
| Missing |  |  | 1 (6.7%) |  |
| **Household Income** |  |  |  |  |
| <$50,000 | 3 (11.1%) | 10 (27.8%) | 2 (13.3%) | 0.39 |
| $50,000 - $99,999 | 11 (40.7%) | 15 (41.7%) | 5 (33.3%) |  |
| > $100,000 | 12 (44.4%) | 10 (27.8%) | 7 (46.7%) |  |
| Missing | 1 (3.7%) | 1 (2.8%) | 1 (6.7%) |  |
| **Batch** |  |  |  |  |
| 1 | 23 (85.2%) | 31 (86.1%) | 12 (80.0%) | 0.84 |
| 2 | 4 (14.8%) | 5 (13.9%) | 3 (20.0%) |  |
| **Study Site** |  |  |  |  |
| Drexel | 10 (37.0%) | 4 (11.1%) | 4 (26.7%) | 0.01 |
| Johns Hopkins | 1 (3.70%) | 13 (36.1%) | 5 (33.3%) |  |
| Kaiser | 9 (33.3%) | 15 (41.7%) | 4 (26.7%) |  |
| UC Davis | 7 (25.9%) | 4 (11.1%) | 2 (13.3%) |  |
| **Cell Type Percent** |  |  |  |  |
| Granulocyte | 74.9 (5.62) | 72.6 (7.36) | 73.8 (6.56) | 0.41 |
| CD8+ Tcell | 6.13 (2.52) | 6.52 (2.47) | 5.76 (4.09) | 0.68 |
| CD4+ Tcell | 6.60 (3.85) | 7.28 (4.41) | 8.69 (3.72) | 0.29 |
| NK Cell | 0.91 (1.71) | 1.35 (2.15) | 0.44 (0.67) | 0.25 |
| Bcell | 3.35 (1.78) | 3.37 (2.12) | 3.21 (1.90) | 0.97 |
| Monocyte | 7.55 (2.20) | 8.25 (2.81) | 7.93 (3.14) | 0.60 |

**Supplementary Table 3. Fetal side placenta study sample descriptive statistics by Baby Siblings Research Consortium algorithm typically developing, non-typically developing, and Autism Spectrum Disorder (ASD) categorization. Mean (SD) for continuous covariates and N(%) for categorical covariates.**

| **Covariate** | **Typically developing**  **N=35** | **Non-typically developing**  **N=48** | **ASD**  **N=18** | **P** |
| --- | --- | --- | --- | --- |
| **Sibling Sex** |  |  |  |  |
| Female | 18 (51.4%) | 24 (50.0%) | 1 (5.56%) | 0.002 |
| Male | 17 (48.6%) | 24 (50.0%) | 17 (94.4%) |  |
| **Maternal Age** |  |  |  |  |
| Continuous | 34.8 (4.07) | 33.3 (4.77) | 34.1 (3.94) | 0.307 |
| **Paternal Age** |  |  |  |  |
| Continuous | 35.8 (6.16) | 35.8 (6.15) | 35.1 (5.99) | 0.897 |
| **Race/Ethnicity** |  |  |  |  |
| Non-Hispanic White | 19 (54.3%) | 14 (29.2%) | 8 (44.4%) | 0.132 |
| Non-Hispanic Black | 1 (2.9%) | 6 (12.5%) | 2 (11.1%) |  |
| Hispanic/Latino | 2 (5.7%) | 11 (22.9%) | 4 (22.2%) |  |
| Other | 8 (22.9%) | 11 (22.9%) | 4 (22.2%) |  |
| Missing | 5 (14.3%) | 6 (12.5%) |  |  |
| **Maternal Education** |  |  |  |  |
| High school or Less | 2 (5.71%) | 5 (10.4%) | 3 (16.7%) | 0.081 |
| Some college | 7 (20.0%) | 19 (39.6%) | 6 (33.3%) |  |
| Bachelors Degree | 8 (22.9%) | 14 (29.2%) | 3 (16.7%) |  |
| Graduate Degree | 18 (51.4%) | 10 (20.8%) | 4 (22.2%) |  |
| Missing |  |  | 2 (11.1%) |  |
| **Household Income** |  |  |  |  |
| <$50,000 | 8 (22.9%) | 16 (33.3%) | 5 (27.8%) | 0.834 |
| $50,000 - $99,999 | 13 (37.1%) | 16 (33.3%) | 6 (33.3%) |  |
| > $100,000 | 13 (37.1%) | 13 (27.1%) | 5 (27.8%) |  |
| Missing | 1 (2.9%) | 3 (6.3%) | 2 (11.1%) |  |
| **Batch** |  |  |  |  |
| 1 | 35 (100%) | 48 (100%) | 18 (100%) | - |
| **Study Site** |  |  |  |  |
| Drexel | 18 (51.4%) | 7 (14.6%) | 4 (22.2%) | <0.001 |
| Johns Hopkins | 3 (8.57%) | 18 (37.5%) | 4 (22.2%) |  |
| Kaiser | 8 (22.9%) | 18 (37.5%) | 3 (16.7%) |  |
| UC Davis | 6 (17.1%) | 5 (10.4%) | 7 (38.9%) |  |
| **Cell Type Percent** |  |  |  |  |
| Trophoblasts | 14.1 (4.98) | 14.4 (5.76) | 14.1 (6.63) | 0.949 |
| Syncytiotrophoblast | 65.6 (9.30) | 65.0 (8.36) | 68.2 (10.1) | 0.442 |
| Stromal | 10.3 (4.59) | 10.2 (3.50) | 9.15 (2.61) | 0.539 |
| Endothelial | 5.74 (2.36) | 6.32 (2.99) | 5.48 (2.49) | 0.451 |
| Hofbauer | 2.97 (1.97) | 2.73 (1.34) | 2.08 (1.21) | 0.152 |
| nRBC | 1.37 (2.48) | 1.33 (1.10) | 0.98 (0.91) | 0.701 |

**Supplementary Table 4. Maternal side placenta study sample descriptive statistics by Baby Siblings Research Consortium algorithm typically developing, non-typically developing, and Autism Spectrum Disorder (ASD) categorization. Mean (SD) for continuous covariates and N(%) for categorical covariates.**

| **Covariate** | **Typically developing**  **N=38** | **Non-typically developing**  **N=48** | **ASD**  **N=15** | **P** |
| --- | --- | --- | --- | --- |
| **Sibling Sex** |  |  |  |  |
| Female | 19 (50.0%) | 28 (58.3%) | 3 (20.0%) | 0.035 |
| Male | 19 (50.0%) | 20 (41.7%) | 12 (80.0%) |  |
| **Maternal Age** |  |  |  |  |
| Continuous | 34.7 (4.16) | 32.8 (4.65) | 34.4 (4.53) | 0.139 |
| **Paternal Age** |  |  |  |  |
| Continuous | 35.9 (5.77) | 35.2 (6.37) | 35.1 (5.19) | 0.840 |
| **Race/Ethnicity** |  |  |  |  |
| Non-Hispanic White | 25 (65.8%) | 17 (35.4%) | 9 (60.0%) | 0.033 |
| Non-Hispanic Black | 1 (2.6%) | 6 (12.5%) | 2 (13.3%) |  |
| Hispanic/Latino | 2 (5.3%) | 10 (20.8%) | 2 (13.3%) |  |
| Other | 7 (18.4%) | 12 (25.0%) | 1 (6.7%) |  |
| Missing | 3 (7.9%) | 3 (6.3%) | 1 (6.7%) |  |
| **Maternal Education** |  |  |  |  |
| High school or Less | 2 (5.26%) | 5 (10.4%) | 2 (13.3%) | 0.021 |
| Some college | 6 (15.8%) | 21 (43.8%) | 6 (40.0%) |  |
| Bachelors Degree | 13 (34.2%) | 15 (31.2%) | 4 (26.7%) |  |
| Graduate Degree | 17 (44.7%) | 7 (14.6%) | 3 (20.0%) |  |
| **Household Income** |  |  |  |  |
| <$50,000 | 6 (15.8%) | 18 (37.5%) | 4 (26.7%) | 0.092 |
| $50,000 - $99,999 | 12 (31.6%) | 14 (29.2%) | 7 (46.7%) |  |
| > $100,000 | 19 (50.0%) | 13 (27.1%) | 4 (26.7%) |  |
| Missing | 1 (2.6%) | 3 (6.3%) |  |  |
| **Batch** |  |  |  |  |
| 1 | 38 (100%) | 48 (100%) | 15 (100%) | - |
| **Study Site** |  |  |  |  |
| Drexel | 18 (47.4%) | 8 (16.7%) | 4 (26.7%) | 0.004 |
| Johns Hopkins | 3 (7.89%) | 14 (29.2%) | 2 (13.3%) |  |
| Kaiser | 9 (23.7%) | 20 (41.7%) | 3 (20.0%) |  |
| UC Davis | 8 (21.1%) | 6 (12.5%) | 6 (40.0%) |  |
| **Cell Type Percent** |  |  |  |  |
| Trophoblasts | 18.5 (5.21) | 17.8 (6.07) | 18.6 (5.22) | 0.828 |
| Syncytiotrophoblast | 59.9 (9.22) | 59.4 (9.42) | 61.1 (7.88) | 0.812 |
| Stromal | 8.92 (2.41) | 9.81 (3.77) | 7.96 (2.98) | 0.124 |
| Endothelial | 7.49 (2.66) | 8.10 (3.29) | 6.61 (2.44) | 0.219 |
| Hofbauer | 3.27 (1.85) | 3.01 (1.80) | 3.13 (2.52) | 0.828 |
| nRBC | 1.93 (1.43) | 1.90 (1.75) | 2.61 (4.67) | 0.557 |

| **Supplementary Table 6.** Number and percent of CpG sites with ASD associations having estimated effect sizes <0 (hypomethylated) or >0 (hypermethylated) among nominally associated sites (*P<*0.05). | | | | | |
| --- | --- | --- | --- | --- | --- |
| Tissue | Hypomethylated | | | Hypermethylated | |
|  | N | | % | N | % |
| Cord Blood | 6177 | 31.2 | | 13624 | 68.8 |
| Maternal Blood – Early Pregnancy | 6796 | 33.23 | | 13658 | 66.77 |
| Maternal Blood – Late Pregnancy | 5035 | 27.7 | | 13145 | 72.3 |
| Placenta –  Fetal Side | 18224 | 77.15 | | 5397 | 22.85 |
| Placenta –  Maternal Side | 13879 | 55.75 | | 11017 | 44.25 |

**Supplementary Table 7.** Single site results for surrogate variable models comparing autism spectrum disorder to typically developing in cord blood, early pregnancy maternal blood, late pregnancy maternal blood, fetal side placenta, and maternal side placenta.

<https://drive.google.com/file/d/1CHTvERyq_YxTCw5YIGDwLycHOQYcztxI/view?usp=sharing>

**Supplementary Table 8.** Single site results for known covariate models comparing autism spectrum disorder to typically developing in cord blood, early pregnancy maternal blood, late pregnancy maternal blood, fetal side placenta, and maternal side placenta.

<https://drive.google.com/file/d/1n0Zx8k8bUe-iveGsQgONWiiicFV_lcRI/view?usp=sharing>

**Supplementary Table 10.** Single site results for surrogate variable models comparing non-typically developing to typically developing in cord blood, early pregnancy maternal blood, late pregnancy maternal blood, fetal side placenta, and maternal side placenta.

<https://drive.google.com/file/d/1_yGLBHjJgbLiu8kokZ9NogY39Y5-4d2X/view?usp=sharing>

**Supplementary Table 11.** Single site results for known covariate models comparing non-typically developing to typically developing in cord blood, early pregnancy maternal blood, late pregnancy maternal blood, fetal side placenta, and maternal side placenta.

<https://drive.google.com/file/d/1n0WceTrHvAhyH_kIxNY3IdN2BhA1dCnk/view?usp=sharing>
